# Comparing apoplastic root barrier formation and morphology in six crop species cultivated in soil vs. hydroponics

**DOI:** 10.1007/s00425-025-04862-3

**Published:** 2025-11-01

**Authors:** Jorge Carvajal, Kiran Suresh, Sabarna Bhattacharyya, Viktoria V. Zeisler-Diehl, Tobias Wojciechowski, Lukas Schreiber

**Affiliations:** 1 Department of Ecophysiology, Institute of Cellular and Molecular Botany, University of Bonn, Kirschallee 1, 53115 Bonn, Germany; 2 Plant Cell Biology, Institute of Cellular and Molecular Botany, University of Bonn, Kirschallee 1, 53115 Bonn, Germany; 3 Plant Sciences (IBG-2), Forschungszentrum Jülich GmbH, 52425 Jülich, Germany

**Keywords:** Cultivation condition, Differential gene expression, Lignin, Root system architecture, Suberin

## Abstract

**Main conclusion:**

Cultivation medium affects apoplastic root barrier formation, gene expression, and morphology across crops, showing that soil growth compared to hydroponics strengthens suberization and lignification while altering plant structural traits.

**Abstract:**

Hydroponic cultivation is commonly used in plant physiology studies; however, studies involving soil are rare. The response of 3 monocotyledonous and 3 dicotyledonous species to cultivation in soil compared with that to cultivation in hydroponic solution was investigated along with the quantification of relevant morphological parameters. The root anatomy was studied with the help of histochemical and microscopic analyses. Root suberin and lignin content were quantified via gas chromatography and mass spectrometry. Transcriptional changes were assessed via RNA-Seq analyses which compared the two growth conditions of barley plants. The results revealed that the plants of all the species cultivated in soil presented significantly longer roots and higher suberin and lignin contents. The above-ground organs of the plants grown in the hydroponic solution presented greater biomass accumulation, with greater shoot dry weights and leaf surface areas. We conclude that across a range of crop genera, the different physicochemical characteristics of the two cultivation media have a pronounced influence on plant morphology, root system architecture, and apoplastic barrier formation.

**Supplementary Information:**

The online version contains supplementary material available at 10.1007/s00425-025-04862-3.

## Introduction

The main function of the root system is water and solute uptake. Uptake is highly dependent on anatomical structure, growth conditions, and plant age. It is best described by the composite transport model. There are three major pathways for water and solute transport in roots: (i) the apoplastic pathway (cell walls), (ii) the symplastic pathway, and (iii) the transcellular pathway. The last two pathways are also known as the cell-to-cell pathway, which can be regulated by aquaporins. The apoplastic pathway can be blocked by the formation of suberin lamellae and Casparian strips in endodermal and exodermal cell walls (Ranathunge et al. 2017; Kreszies et al. 2020).

Suberin found in the suberin lamellae in roots consists of polyaliphatic and polyaromatic domains cross-linked via ester bonds. The aliphatic monomers are primary alcohols, fatty acids, α,ω-dicarboxylic acids (diacids), and ω-hydroxy acids (ω-OH acids), whereas the aromatic monomers include coumaric and ferulic acids (Franke and Schreiber 2007; Graça 2015; Ranathunge et al. 2017). Casparian strips are mainly composed of (poly) phenolics (Schreiber 1996; Naseer et al. 2012). Suberin, consisting of both aliphatic and aromatic monomers, as well has lignin have been identified in certain species as a relevant fraction of Casparian strips (Schreiber et al. 1999; Thomas et al. 2007). Lignin monomers are composed of three types of monolignols: syringyl, guaiacyl, and *p*-hydroxyphenyl alcohols, which make it an aromatic polymer (Liu et al. 2018).

The deposition of both biopolymers in roots has been shown to vary in response to different abiotic stresses, such as water deficit, salinity, or hypoxia (Krishnamurthy et al. 2009; Moura et al. 2010; Abiko et al. 2012; Dos Santos et al. 2015; Kotula et al. 2017; Kreszies et al. 2019; Suresh et al. 2024).

In most studies investigating suberin and lignin deposition in roots, hydroponically cultivated plants are used. In this technique, plants are grown in a nutrient solution without the use of a solid medium to provide mechanical support. This cultivation system is artificial but has multiple advantages that have made it a useful tool in plant physiology studies. By modifying the nutrient mixture, several abiotic stresses can be studied: by adding PEG8000, osmotic stress can be induced, mimicking water stress, reducing aeration can induce hypoxia, and modifying the nutrient composition can be used to study the effects of deficiencies or excesses of macro- and micronutrients and toxic compounds (Enstone and Peterson 2005; Krishnamurthy et al. 2011; Abiko et al. 2012; Osmolovskaya et al. 2018; Armand et al. 2019; Kreszies et al. 2019; Melino et al. 2021).

The growth rate and yield of hydroponically grown plants are expected to be greater than those of plants that are traditionally grown in soil (Sardare and Admane 2013; Sharma et al. 2018; Gaikwad 2020). As roots are directly suspended in a nutrient solution, water and nutrient uptake are more efficient, and most of the energy is directed toward the growth of above-ground organs (Sardare and Admane 2013; Lei and Engeseth 2021). Therefore, the use of hydroponic systems has increased as an alternative to conventional soil cultivation. Under controlled greenhouse conditions, hydroponic cultivation allows sustainable crop production throughout the year (Geilfus 2019). Nevertheless, soil-based cultivation systems should provide a closer similarity between experimental conditions and those experienced in nature and agriculture. Different cultivation conditions can create significantly different environments that can affect root structure, growth, and physiology. Recent results have shown that apoplastic root formation, plant morphology, and gene expression differ in barley plants cultivated in soil, both under control conditions and different water stress conditions (Suresh et al. 2024).

In this study, the effects of cultivation in soil and hydroponics on apoplastic barrier development and plant morphology in six different plant species under control conditions have been investigated. Suberin and lignin deposition along the root was investigated via microscopy and subsequently chemically analyzed via gas chromatography and mass spectrometry. Changes in the gene expression patterns of barley in the two cultivation media were investigated in detail. We hypothesize that root and shoot growth, as well as root apoplastic barrier development, differ between soil cultivation and hydroponic cultivation.

## Materials and methods

### Plant material and growth conditions

The experiments were performed with six crop species: three monocotyledonous (*Hordeum vulgare* ‘Scarlett’ [barley], *Triticum aestivum* ‘Akteur’ [wheat], and *Zea mays ‘*Falcone’ [corn]) and three dicotyledonous (*Phaseolus vulgaris* ‘Saxa’ [broad bean], *Solanum lycopersicum* ‘Golden Queen’ [tomato], and *Vigna radiata*, unknown variety [mung bean]). Seeds of all the species were soaked in water supplemented with 50 mM gibberellic acid (3-GAA) for 24 h to ensure proper and uniform germination. Seeds used for hydroponic cultivation were germinated in the dark at 25 °C and covered with wet paper towels. The monocotyledonous species were germinated for 3 days, mung bean and broad bean for 5 days, and tomato for 7 days. Post-germination, seedlings with uniform root and shoot germination were transferred into a hydroponic system containing half-strength Hoagland solution with a continuous oxygen supply.

Seeds from the soil experiments were directly sown into the sieved substrate (Soil Type I; Einheitserde, Sinntal-Altengronau, Germany) filled in rhizotrons with the following measures: 80 cm in height, 30 cm wide, and an inner width of 2.4 cm, consisting of a transparent polycarbonate plate covered with an opaque panel. In addition, the transparent side is always positioned facing downward to prevent the penetration of light. The inclination angle of the rhizotrons was adjusted to 43° (Nagel et al. 2012). For both cultivation treatments, the experiment was performed under control conditions, with the soil being watered with 400 ml of tap water three times per week. Plant cultivation for both conditions took place in a growth chamber under long-day conditions (16 h:8 h, light:dark), air temperatures of 23 °C (day) and 20 °C (night), and relative air humidities ranging from 50 to 65%. The monocotyledonous species were cultivated for 12 days, mung bean and broad bean for 15 days, and tomato for 25 days. Varying harvest times for the different species were chosen to obtain comparable plant developmental states. All the plants were cultivated until they developed their first two or three fully mature leaves. After harvesting, the root and shoot lengths were measured, and the samples were dried for 1 week in a 60 °C oven. The samples were subsequently weighed, and the root/shoot ratio for each species under each cultivation condition was calculated. The leaves of each plant were scanned to calculate the leaf surface area. The water potentials for both cultivation conditions were measured. The nutrient solution, measured with a freezing point osmometer (Model 3000, Gonotec, Berlin, Germany), had a water potential of – 0.016 ± 0.002 MPa. The soil, measured with a soil water potential instrument (WP4C, Meter Group, München, Germany), had a water potential of – 0.06 ± 0.03 MPa.

### Histochemical detection of suberin lamellae deposition and lignified tissues in roots

The root sample was segmented into 1 cm increments from the base to the tip and stored in 1.5 ml Eppendorf tubes with fixation solution. Using a cryostat microtome (Microm HM 500 M, Microm International, Walldorf, Germany), approximately 50 µm-thick cross-sections were prepared. To detect suberin deposition over root length, cross-sections were stained with 0.01% (w/v) lipophilic fluorol yellow 088 for 1 h (Brundrett et al. 1991; Kreszies et al. 2019). Lignin was stained with 1% (w/v) safranin red for 10 min, rinsed with ethanol to remove excess stain, and subsequently stained with 1% (w/v) astra blue for 10 min (Suresh et al. 2024). These two dye solutions are used for differential staining of lignified tissues: safranin red stains lignin red, whereas astra blue stains cellulose blue. The use of both allows the observation of lignified structures and the acquisition of contrasting images via light microscopy (Vazquez-Cooz and Meyer 2002; Novikov and Sup-Novikova 2021). The root samples were cross-sectioned at representative relative lengths corresponding to previously characterized developmental zones, with 0% of the relative root length defined as the root tip and 100% as the root base (Kreszies et al. 2019). For suberin staining and subsequent chemical analysis, the whole root was investigated; for lignin, only the top 50% was analyzed due to sample amount limitations. Cross-sections were analyzed via fluorescence microscopy via an ultraviolet (UV) filter set (excitation filter BP 365, dichroic mirror FT 395, barrier filter LP 397; Zeiss, Oberkochen, Germany). Images were obtained with a Canon EOS 600D camera at ISO 100–400.

### Chemical analysis of suberin and lignin

The roots were divided into three zones (A, B, and C). Zone A (0–25% of the total root length) corresponds to the youngest part of the root, including the root apex. Zone B (25–50%) is the transition zone, and Zone C (50–100%) is the mature part of the root, as described previously (Suresh et al. 2024). The observed cross-sections are expressed as relative length percentages of the whole roots, with 0% corresponding to the root tip and 100% to the root base. For each growth condition, three independent biological replicates were harvested. Every replicate consisted of 10 segments from each root zone pooled together from 4 to 5 plants. The samples were enzymatically digested for 3 weeks with 0.5% (w/v) cellulase and 0.5% (w/v) pectinase at room temperature under constant shaking (Zeier and Schreiber 1998). The digestive enzyme mixture was replaced every 3–5 days. Finally, the roots were washed in borate buffer (pH 9, 0.01 M) for 24 h and then transferred to 1:1 (v/v) chloroform:methanol for soluble lipid extraction under continuous shaking for 2 weeks, followed by washing in deionized water. No mechanical separation of endodermal and vascular tissue after the enzymatic digestion was performed. Before chemical analyses, the samples were dried, weighed, and cut into fine pieces.

For suberin analysis, the samples were transesterified with BF_3_-methanol to release suberin monomers (Kolattukudy and Agrawal 1974) and complemented with 10 µg of internal standard (C_32_—dotriacontane, Fluka) for single-monomer quantification. Suberin monomers were extracted three times via chloroform; the sample volume was reduced under a gentle stream of nitrogen and derivatized with 20 µl of pyridine (Sigma Aldrich) and 20 µl of BSTFA [N, O-bis (trimethylsilyl)-trifluoroacetamide] (Baales et al. 2021). The monomers were quantitatively analyzed via GC-FID with a splitter-injection system (Straube et al. 2025). Mass spectrometric identification was performed as described earlier via an in-house-created library (Schreiber et al. 2005). For lignin analysis, samples were incubated in dioxane-ethanethiol in the presence of BF_3_ for 4 h at 105 °C with regular shaking. This reaction is known as thioacidolysis and results in the depolymerization of lignin. After ethyl acetate extraction of the monomers, the sample volume was reduced completely, acetone was added twice to ensure residual water evaporation, and the samples were resuspended in 100 µl of chloroform. The samples were subsequently derivatized with 20 µl of pyridine and 100 µl of BSA [N, O-bis(trimethylsilyl)acetamide] and analyzed via gas chromatography and mass spectrometry according to previously described methods (Lapierre et al. 1985; Reale et al. 2004; Robinson and Mansfield 2009; Foster et al. 2010). This analysis was performed for the three root zones in barley and only for Zone C in the other species. Suberin amounts refer to the endodermal/exodermal (corn, tomato) surface areas of each zone. The lignin content was calculated based on the exodermal surface area. In the monocotyledonous species, it was calculated on the basis of a cylindrical shape: *A* = 2π*rL* (*r*, radius; *L*, length of the individual root zone). For the dicotyledonous species, a truncated cone shape was assumed: *A* = *π(R* + *r√((R—r)*^*2*^ + *h*^*2*^*)* (*R*, endodermis [broad and mung bean]/exodermis [tomato] radius at the basal side of the root zone; *r*, radius at the apical side of the root zone; *h*, length of the root zone).

### Comparison and meta-analyses of RNA-Seq data

For barley, gene expression was compared between hydroponically grown roots and soil-grown roots. For RNA-Seq data concerning roots grown in soil, reads from a recently published study were used (Suresh et al. 2024) (SRA accession: PRJNA1063280), where Zone A (0–12.5% of root length) was harvested for sequencing. The transcriptomic data obtained from hydroponics Zones A and B (25–37.5%) were paired reads from a previous study (Kreszies et al. 2019) (SRA accession: SRP136092). The raw reads from both studies were subjected to a quality check via FastQC, followed by adapter trimming with cutAdapt (Martin 2011). The processed reads were then aligned with the barley reference genome (EnsemblPlants, http://plants.ensembl.org/Hordeum_vulgare) with Tophat2 with the help of a bowtie index generated from the individual chromosome files (Trapnell et al. 2012). Mapping statistics with the aligned files in BAM format were obtained via SAMtools (Li et al. 2009), and a mapping rate of ≥ 90% was considered the threshold. Read processing and alignment were carried out in a UNIX shell environment on an Ubuntu 18.04 LTS operating system. Using the edgeR package (Robinson et al. 2010) in RStudio, gene count generation, CPM (counts per million) estimation, and differential expression analysis were performed. To test the homogeneity of all the replicates, before differential expression analyses, the samples were grouped in a multidimensional scaling plot (MDS plot) with the Limma package in R (Ritchie et al. 2015). Differential expression analyses were performed with a log_2_FC cutoff of 1 (soil vs. hydroponics) and a false discovery rate (FDR) cutoff of ≤ 5% (Benjamini and Hochberg 1995). Gene Ontology (GO) terms were obtained from the differentially expressed genes (DEGs) via ShinyGO software available online (Ge et al. 2020). Using the BART tool, a homology search of the DEGs against the model organism *Arabidopsis thaliana* (E value cutoff < 1e^−30)^ was performed on https://ics.hutton.ac.uk/barleyrtd/ (Mascher et al. 2017).

### Statistical analysis of chemical and physiological data

Morphological and chemical data were analyzed via one-way analysis of variance (ANOVA) to evaluate differences among groups. Post hoc comparisons were performed via Fisher’s LSD test to identify specific and significant group differences. The results are presented as the means ± standard deviations (SDs). Statistical significance was set at *P* < 0.05. All analyses were conducted via Origin Pro 2021b (OriginLab Corporation).

## Results

### Morphological parameters and root anatomy

**Fig. 1:**
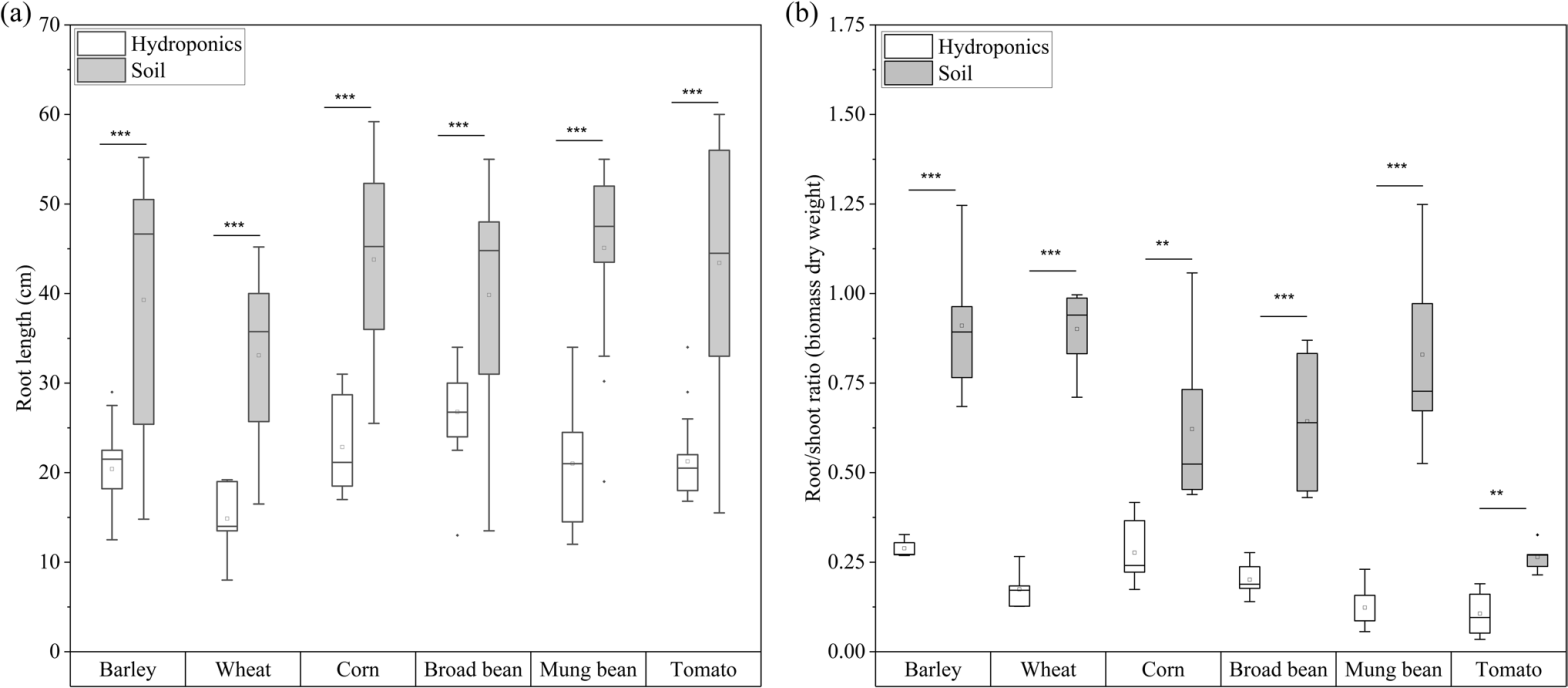
Phenotypic characterization of hydroponically cultivated and soil-grown plants. **a** Average root length (cm). **b** Root/shoot ratios (biomass dry weight). The box ranges from the 25th to the 75th percentiles. The square inside the box represents the mean value. The whiskers range to outliers, and each box represents > 30 individual root lengths. Each crop species was separately analyzed via one-way ANOVA (Fischer’s least significant difference, LSD), with cultivation condition as a factor. Significance: *P* < 0.001**; *P* < 0.01**; *P* < 0.05*; *n* = 3 replicates; *n.s.* not significant

For the six species, plants grown in soil had significantly longer roots (on average, two times greater) than those cultivated in hydroponic solution (Fig. 1a). The root dry weight was significantly higher in soil-grown plants than in hydroponically cultivated plants (Fig. S1a). Consequently, the root/shoot ratios for the soil-grown species were significantly larger, with an increase of two- to threefold (Fig. 1b).

In contrast, hydroponically grown plants showed greater above-ground biomass accumulation. All the species presented greater shoot dry weights when cultivated in hydroponic solution (Fig. S1b). For dicotyledonous species, this trend was also visible when the total leaf surface area of hydroponically grown plants was compared with those cultivated in soil. All of them had significantly greater total leaf surface areas when grown hydroponically. However, this was not observed for monocotyledonous species (Fig. S1c). Barley and wheat had a greater average total leaf surface area when grown in soil, but this was due to a difference in the number of leaves on which the plants developed. Three leaves developed when cultivated in soil, but only two developed when cultivated hydroponically (Fig. S1d).

### Histochemical detection of suberin and lignin

**Fig. 2:**
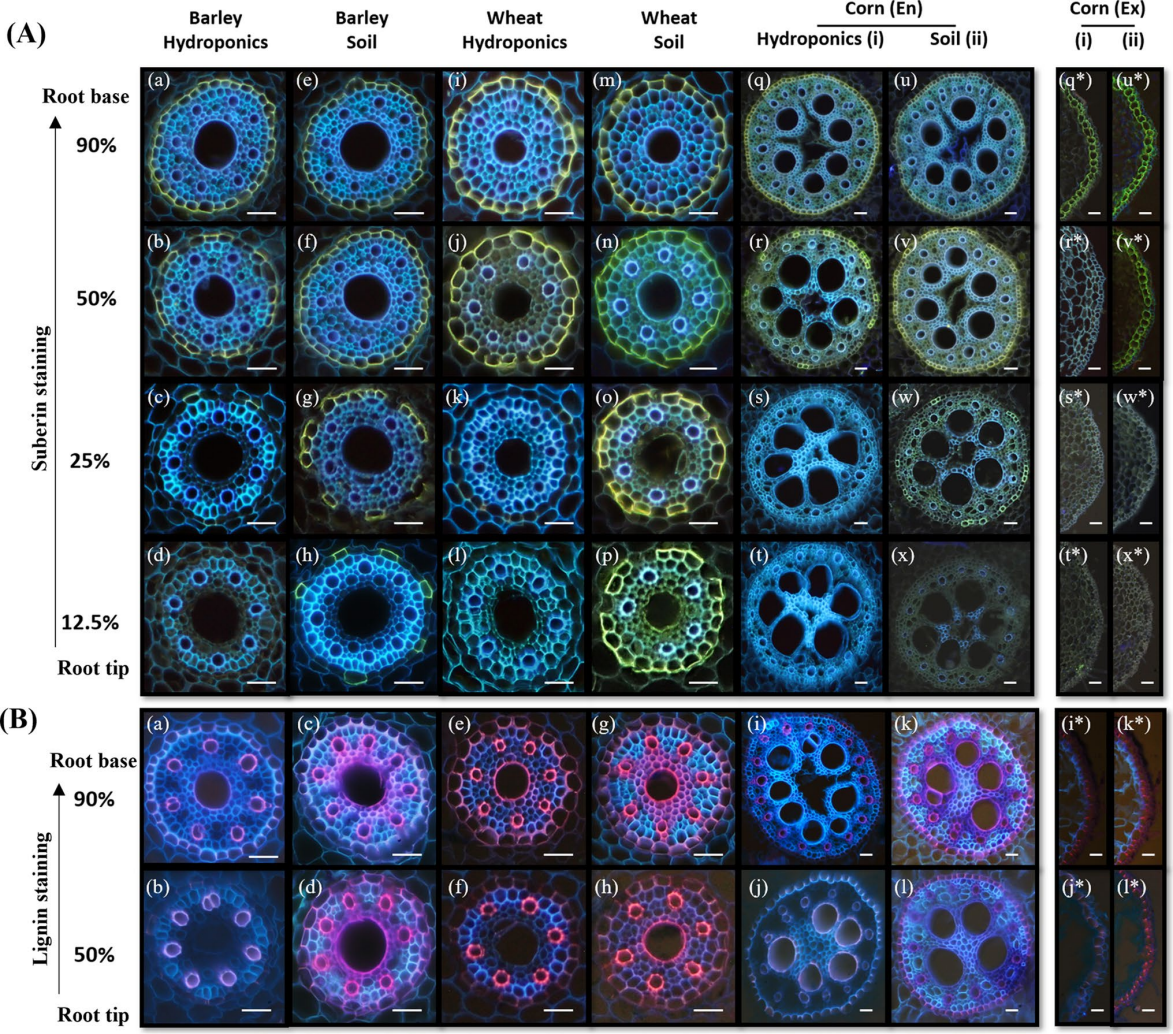
Histochemical analyses of root suberization and lignification in monocot species. **A** Suberin-lamellae development in roots of plants grown in hydroponics and soil conditions. The samples were stained with fluorol yellow 088, and the presence of suberin lamellae was indicated by bright yellow fluorescence. The endodermal suberization is shown for all three monocots **(a–x)** and the exodermal suberization is shown for only corn (*). At a distance of 90%, all the cells are completely suberized (a, e, i, m, q, u, q*, u*). At 50% of root length, all the cells are suberized (b, f, j, n, v, v*) except for both the endodermis and exodermis of corn roots grown in hydroponic solution (r, r*). At a distance of 25%, partially suberized **(c, k)** or no suberized cells (s, s*, w*) can be seen for hydroponically cultivated roots. The soil-grown roots have higher suberization **(g, o, w)**. At 12.5%, only soil-grown roots of barley and wheat have some suberized cells **(h, p)**. Overall, the suberization is stronger in soil-grown roots compared to hydroponically cultivated roots. **B** Lignification of roots grown in hydroponics and soil conditions for monocots. The root sections are stained with safranin red and counter-stained with astra blue; lignified tissues are red, and cellulose is blue-colored. At a distance of 90%, the inner side of the endodermis (En) and Casparian strips is lignified **(a, c, e, g, i, k)**, and protoxylems are lignified. Some metaxylems are lignified **(a, c, d, e, g, h, k)** and most of them are found in soil-grown roots. Corn roots have lignification of Casparian strips in the exodermis (Ex) (i*, k*, l*). At 50% of root length in corn, Casparian strips are faintly stained for hydroponically grown roots (j*). Soil-grown roots show lignification of the cortical cells **(c, d, g, h)** and inner walls of the endodermis **(a, c, d, g, h, k)**. i = hydroponics; ii = soil-grown; En = endodermis; Ex, * = exodermis; Scale bars = 50 µm

**Fig. 3:**
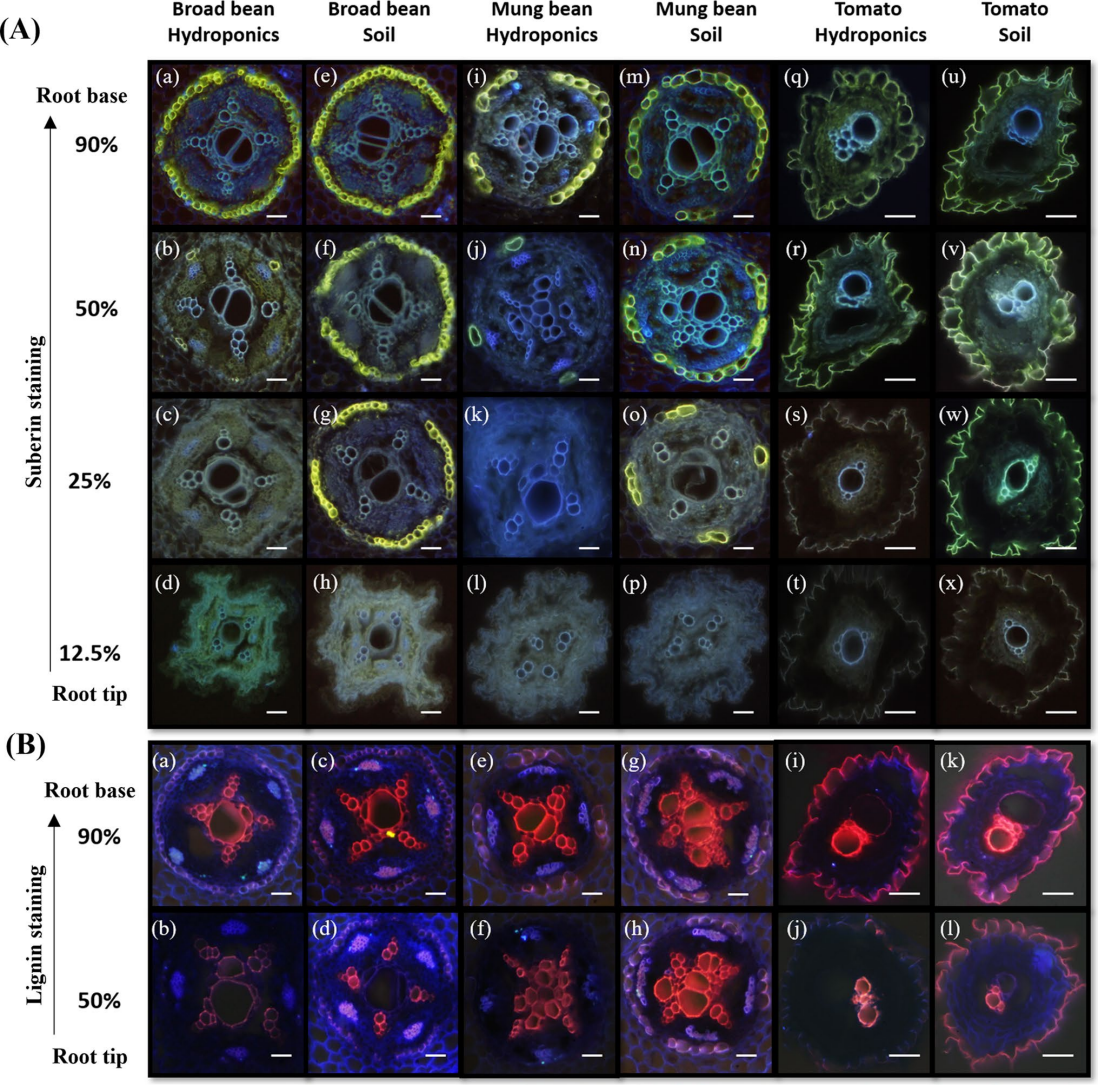
Histochemical analyses of root suberization and lignification in dicot species. **A** After staining with fluorol yellow 088, the presence of suberin lamellae is indicated by bright yellow fluorescence. Endodermal suberization is shown for broad bean and mung bean **(a** to **p)**, and exodermal suberization for tomato **(q** to **x)**. At a distance of 90%, endo- and exodermal cells are almost completely suberized **(a, e, q,** and **u)**, and they are partially suberized for mung bean. At 50%, broad bean and mung bean have greater suberization in the soil **(f, n)** than in hydroponics **(b, j)**. Exodermal cells are completely suberized in tomato cross-sections **(r, v)**. At 25% root length, soil-grown roots are partially suberized **(g, o)**, and there are no suberized cells for hydroponically grown roots **(c, k)**. In soil-grown tomato root cross-sections, the exterior side of the exodermis is strongly suberized **(w)**. At 12.5%, none of the dicot cells are suberized **(d, h, l, p, t, x)**. Overall, suberization is stronger in soil-grown roots than in hydroponically cultivated roots. **B** Root cross-sections are stained with safranin red and counter-stained with astra blue for the detection of lignin; lignified tissues are red, and cellulose is blue. At a distance of 90%, the endodermis of broad bean and mung bean **(a, c, e, g)**, and tomato exodermis are lignified **(i, k)**. At the 50% section, endodermal cells have patchy lignification for soil-grown roots **(d, h)** and complete lignification for tomato exodermis **(l)**. Phloem wall lignification can be observed in soil-grown roots **(c, d, g, h)**. Almost all the walls of the dicot metaxylem and protoxylem are lignified in both hydroponics (faintly stained) and soil-grown (brightly stained) dicots in the basal root region. Scale bars = 50 µm

Histochemical analyses clearly indicated that both suberization and lignification were much stronger in the soil-grown plants than in those cultivated hydroponically (Figs. 2 and 3). The suberin–lamellar development of roots grown hydroponically and the soil conditions for monocotyledonous roots are shown in Fig. 2A. In barley and wheat, the onset of suberization was less than 12.5% of the root length in the soil (Fig. 2A h,p), whereas in hydroponics, partial suberization started at approximately 25% of the root length or later (Fig. 2A c,k). In corn, suberin lamellae were observed in the endodermis as well as in the exodermis (Fig. 2A q to x and q* to x*). Suberized cells of the endodermis were observed at approximately 25% of the root length in soil and only at 50% in hydroponics (Fig. 2A w,r). In the exodermis, suberization in soil was very pronounced at 50% of the root length, whereas in hydroponics, exodermal suberization was only visible in the basal part of the root (Fig. 2A q*, r*, u*, v*). Similar trends as those in monocotyledonous species could be observed in dicotyledonous species (Fig. 3). Endodermal cells were already partially suberized at approximately 25% of the root length in soil-cultivated broad bean and mung bean (Fig. 3A g, o, w), whereas in hydroponically grown roots, very few cells were suberized at approximately 50% of the root length (Fig. 3A b, j, r). In tomato roots, suberin lamellae deposition was only observed in the exodermis. Compared with those cultivated in hydroponics, roots grown in soil have an earlier onset of suberization (Fig. 3A s, w). At 12.5%, none of the cells in the dicotyledonous species were suberized (Fig. 3A d, h, l, p, t, x).

In monocotyledonous species, the xylem vessels and endodermal cell walls of roots cultivated in soil presented a greater degree of lignification than those of plants grown hydroponically (Fig. 2B a to l). This trend was also observed in the exodermis of corn (Fig. 2B i* to l*), where plants grown in soil presented a highly stained exodermis along approximately 50% of the root length, but this was not observed in hydroponics. In older basal root zones (90%), the endodermis was more lignified than the lower root zone (50%). For dicotyledonous species, similar trends could be observed. At 50% root length, the endodermis in broad bean (Fig. 3B b, d), mung bean (Fig. 3B f, h), and exodermis in tomato (Fig. 3B j, l) grown in soil was significantly more lignified than the roots grown in hydroponics.

### Chemical analysis of root suberin and lignin contents

**Fig. 4:**
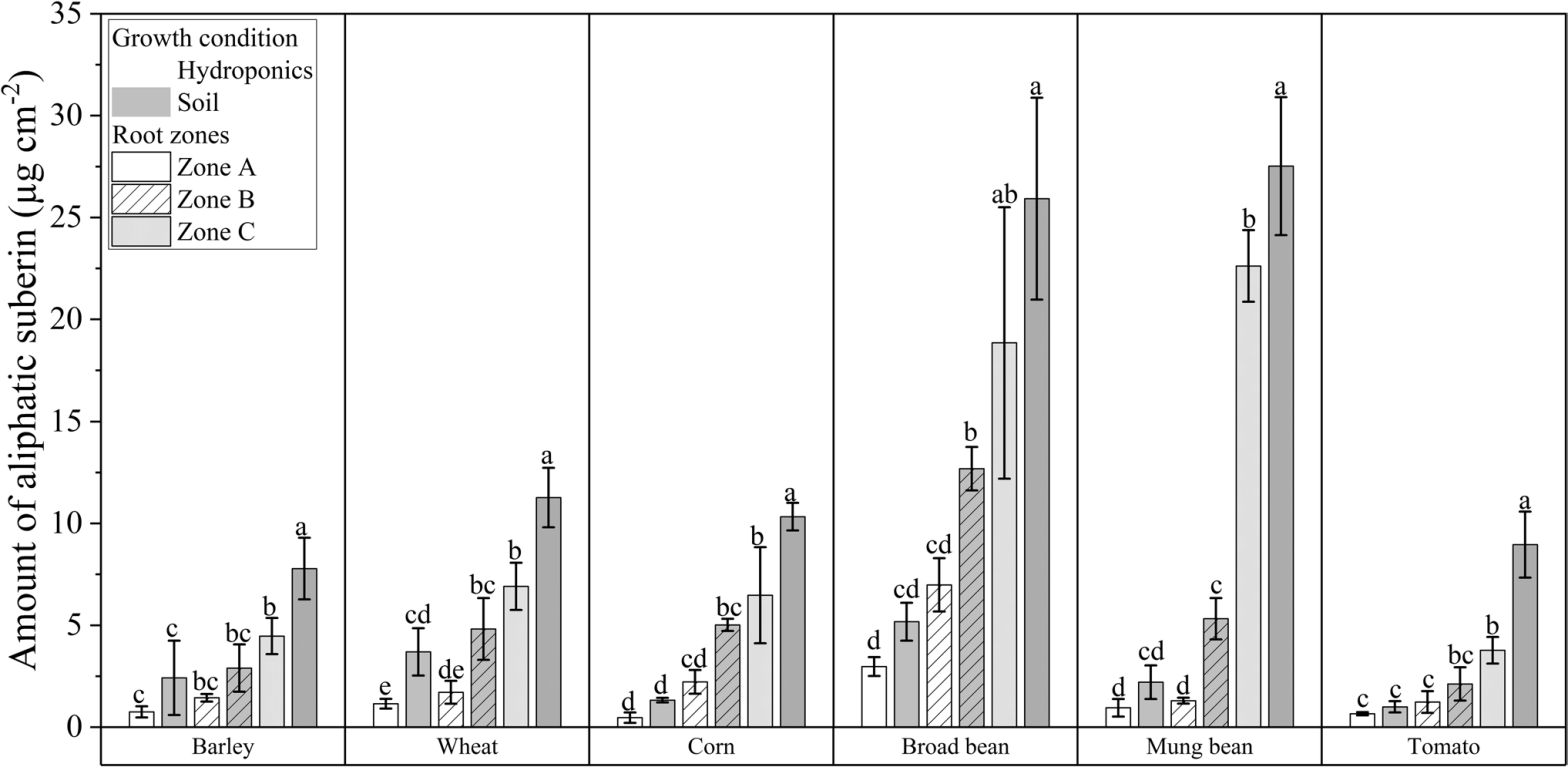
Amounts of aliphatic suberin in roots grown hydroponically and soil conditions. The roots were divided into three root zones from root tip Zones A, B, and C toward the basal part. The bars represent the mean values with a standard deviation of at least three biological replicates (*n* = 3). Different letters indicate significant differences between the means at a significance level of 0.05 according to one-way ANOVA (Fischer’s least significant difference, LSD). The significance level was tested for different root zones within the same species. The amount of suberin increased proportionally across the root zones from A to C under both hydroponic and soil conditions. Most of the soil-grown roots contained more suberin than the roots of hydroponically cultivated plants

**Fig. 5:**
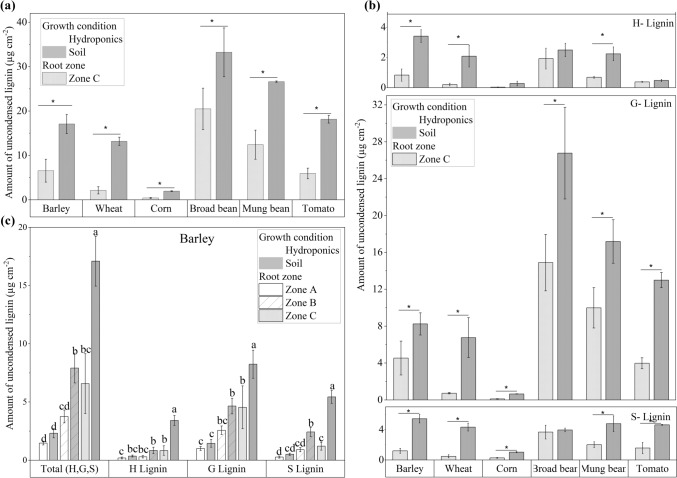
Amounts of uncondensed lignin in roots grown under hydroponic and soil conditions. **a** Total uncondensed lignin in Zones C (H, G, and S) of the six species. **b** Total uncondensed lignin in Zone C is divided into H-, G-, and S-lignin subunits for different crop species. **c** Total and uncondensed lignin in the barley roots of all three root zones. The roots were divided into three root zones, from root tip Zone A, Zone B, and Zone C, toward the basal part. The bars represent the mean values with a standard deviation of three biological replicates (*n* = 3). The significance level was tested between roots grown in hydroponics and cultivated in soil. Different letters as well as indication by an asterisk (*) denote significant differences between the means at a significance level of 0.05 according to one-way ANOVA (Fischer’s least significant difference, LSD). The amount of lignin in soil-grown roots is greater than that in hydroponically cultivated root zones

The results of the chemical analysis fit those of the histochemical analysis of the roots. The suberin and lignin contents were nearly always significantly greater in roots grown in soil than in those grown hydroponically (Figs. 4, 5). The aliphatic suberin is composed of four suberin-characteristic monomer classes: fatty acids (FAs), alcohols (alc), ω-hydroxy acids (ω-OH acids), and α-ω-dicarboxylic acids (diacids) (Fig. S2). The most abundant classes were ω-OH acids and fatty acids. There were statistically significant differences in the aliphatic suberin content between the two cultivation conditions for Zone C among all six species (Fig. 4). In addition, aromatic suberin contents (Fig. S3a) and total suberin contents, which represent the sum of aliphatic and aromatic suberin contents (Fig. S3b), increased over the length of the roots and were greater in the soil than in the hydroponic solution. In addition to suberin, lignin amounts in soil-grown species were also significantly greater than those in hydroponic cultivation (Fig. 5a). The most abundant lignin monomer unit in all six species was G-lignin, followed by S-lignin and H-lignin (Fig. 5b). The amount of all lignin units was significantly greater in the soil-grown roots. In barley, lignin in all three root zones increased significantly along the root from the tip to the base under both conditions (Fig. 5c).

### Differential gene expression analyses between soil-grown and hydroponically grown roots of barley

**Fig. 6:**
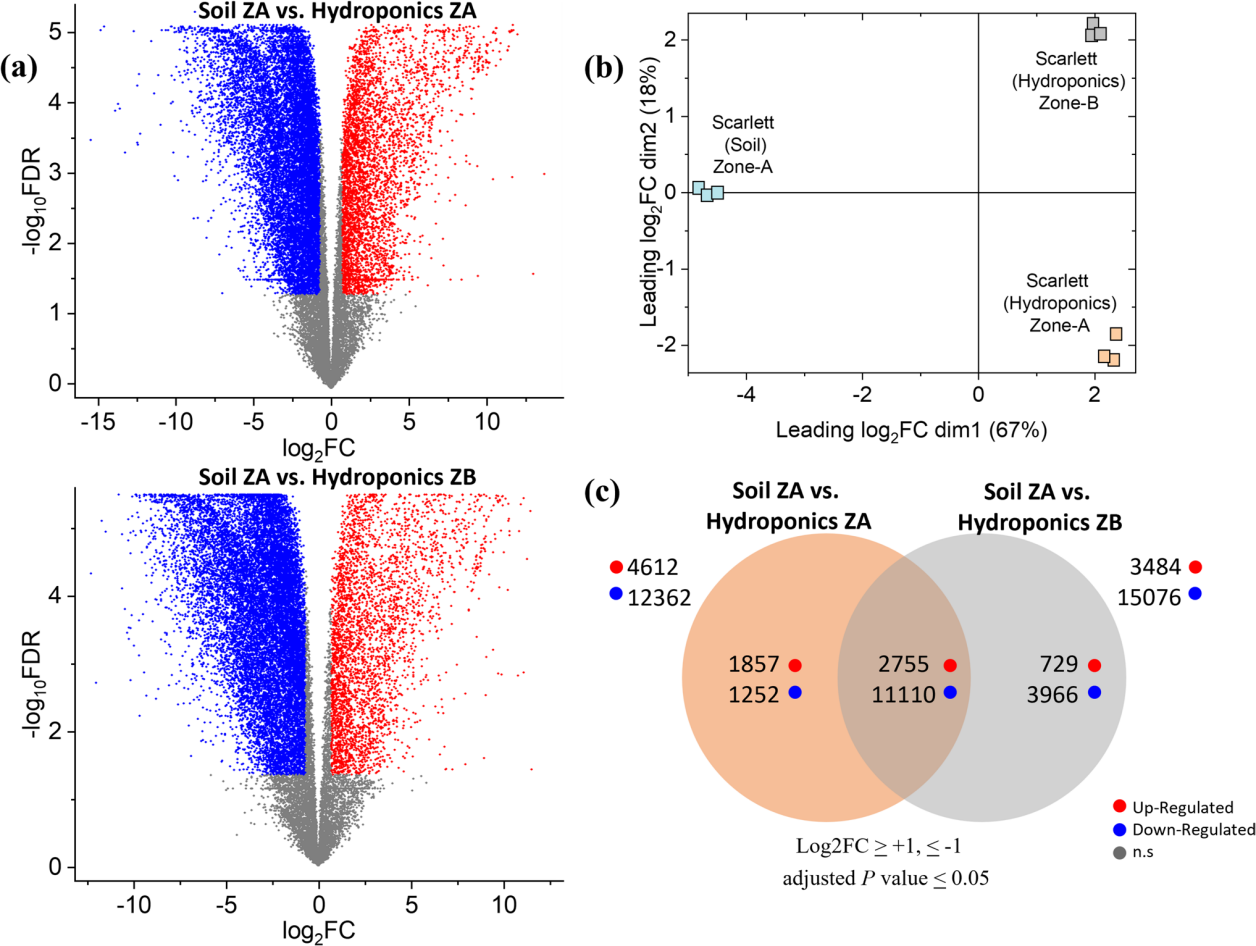
Differentially expressed genes (DEGs) of soil-grown roots of Zone A versus hydroponically grown roots of Zone A and Zone B. **a** Volcano plots of DEGs in soil Zone A versus hydroponics Zone A (upper panel) and soil Zone A versus hydroponics Zone B (lower panel). The X-axis represents the fold change (log2FC) of DEGs (soil vs. hydroponics), whereas the Y-axis represents the statistical significance (log_10_FDR). The length of 0–12.5% of the roots from Zone A soil was compared with data from Kreszies et al. (2019), who used control samples from Zones A and B for expression studies. **b** Multidimensional scaling plot of replicated RNA-sequencing samples. This graph provides a visual representation of sample relationships by spatial arrangements (*n* = 3). **c** Venn diagram representing DEGs (DESeq, Log2FC ≥ 1, ≤ -1, and FDR ≤ 0.05) between soil-grown roots Zone A (ZA) and hydroponically cultivated roots Zone A and B. Among all DEGs, 2755 and 11,110 genes were commonly up- and downregulated in both hydroponic root zones compared with the ZA of soil. Red, blue, and gray dots indicate upregulated, downregulated, and nonsignificant genes, respectively

Paired reads of transcriptomic changes were compared between soil-grown (Suresh et al. 2024) and hydroponically cultivated roots (Kreszies et al. 2019). Preprocessing of reads, including MDS (multidimensional scaling) analyses, confirmed a clear variance-based separation of the three independent biological replicates of soil and hydroponics, thereby reflecting the robustness of the samples used in this study (Fig. 6b). A comparison of RNA-Seq data from hydroponically grown roots of the same cultivar and soil-grown barley roots was performed. A comparison between soil-grown roots (SZA: Zone A, partially suberized endodermis) and hydroponically grown roots (HZA: Zone A, nonsuberized endodermis) revealed a total of 16,974 DEGs, with 12,362 downregulated genes and 4612 upregulated genes. Among these DEGs, 1857 and 1252 DEGs were uniquely up- or downregulated, respectively. Compared with Zone B (partially suberized endodermis), the hydroponically grown roots presented 3484 upregulated and 15,076 downregulated genes, of which 729 were specifically upregulated and 3966 were specifically downregulated. Both comparisons revealed 2755 and 11,110 common up- and downregulations, respectively (Supplementary Table S1). As shown in Fig. 6a and c, comparisons between soil and hydroponic cultivated plants revealed a significantly greater number of downregulated genes than upregulated genes.

**Fig. 7:**
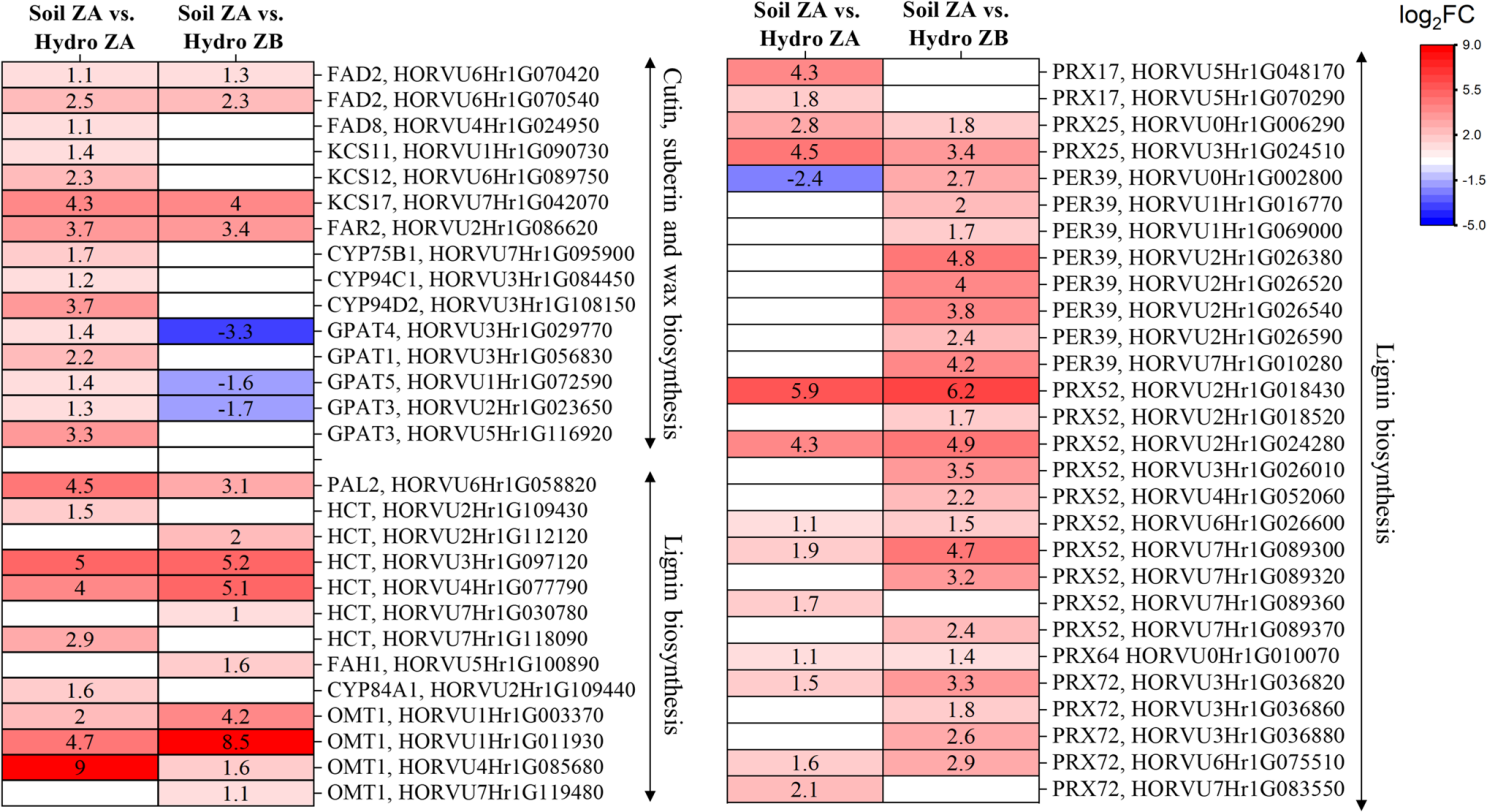
Selected DEGs related to suberin, cutin, cuticular wax and phenylpropanoid biosynthesis were commonly upregulated in soil-grown Zone A roots compared with those in hydroponically grown Zone A and Zone B roots. The empty white cells represent not significantly different (n.s.) genes. Genes with putative barley homologs to their respective Arabidopsis gene ID, identity percentage, and log2FC, descriptions and references are given in Supplementary Table S3

To gain a better understanding of various gene categories that are altered transcriptionally, we performed GO analyses of the various DEGs identified in the comparisons. In Zone A of soil-grown roots (SZA) compared with Zone A of hydroponically grown roots (HZA), DEGs associated with GO terms related to macromolecule synthesis, such as peptide biosynthetic processes, amide biosynthetic processes, and translation, were predominantly upregulated. In contrast, the downregulated DEGs were grouped under GO terms such as macromolecule modification, the cellular protein modification process, and the protein modification process (Supplementary Table S2). Categories of genes previously associated with stress responses in plants, such as aquaporins and genes associated with lignification and suberization, were predominantly upregulated in our analyses (Fig. 7; Fig. S4, Supplementary Table S3). Specifically, the expression of aquaporin genes, including those belonging to the NIP, TIP, and PIP families, was consistently upregulated (Fig. S5, Supplementary Table S4). Genes involved in the biosynthesis of suberin, lignin, cutin, or wax were also largely upregulated. Notably, only the orthologs of GPATs 3, 4, and 5, which are involved in suberin biosynthesis, and one ortholog of PER39 (HORVU0Hr1G002800), which is associated with lignin biosynthesis, were downregulated in comparison with both hydroponic zones (Fig. 7). All other genes involved in lignification and suberization presented a positive log fold change of at least 2 (Fig. 7). The transcriptomic analyses presented here provide strong support for the observations made through microscopy (Figs. 2 and 3) and chemical analysis (Figs. 4 and 5), further validating the relationships between transcriptional changes and the biochemical alterations observed in the roots.

## Discussion

Hydroponic cultivation is widely used in plant physiology because it provides noninvasive access to the root system and allows researchers to monitor growing conditions easily. In contrast, cultivation in soil-filled rhizotrons presents a more field-relevant scenario, stimulating environmental factors such as mechanical resistance and dependence on soil structure and architecture. Although rhizotrons also enable noninvasive root studies, they can be valuable tools for future plant physiology research, offering a closer approximation of natural field conditions (Nagel et al. 2012; Sharma et al. 2018).

### Morphological parameters and root anatomy

Barley plants had an average root length of 22 cm when cultivated in hydroponics, and of 47 cm when cultivated in soil. Soil-cultivated wheat had an average root length of 33 cm, and when cultivated in hydroponics, it was 15 cm. As for corn plants, the average root length in soil was 43 cm, and in hydroponics, it was 24 cm. All these values are consistent with those previously reported in the literature for both soil and hydroponic cultivation (Schreiber et al. 2005; Schneider et al. 2017; Kreszies et al. 2019; Ouyang et al. 2020; Suresh et al. 2024).

In dicots, tomato plants had an average root length of 53 cm in soil and 21 cm in hydroponics. Soil-cultivated broad bean had an average value of 47 cm, and when in hydroponics, the root length was 26 cm. Finally, mung bean had an average root length of 43 cm in soil and 24.5 cm in hydroponics. These values are consistent with the literature for hydroponic cultivation (Calvo-Polanco et al. 2014; Kumar et al. 2016; Ahmad et al. 2019; Hernandez-Espinoza and Barrios-Masias 2020).

In the soil-grown plants, we observed a twofold increase in root length, along with a subsequent consistent increase in the root/shoot ratios (Fig. 1a and b). In contrast, in dicotyledonous species, hydroponically grown plants presented greater shoot biomass accumulation and greater leaf surface area (Fig. S1b and 1c). This discrepancy may be attributed to the different physicochemical and biological characteristics of the two cultivation conditions. Soil is a heterogeneous medium, and its physical, chemical, and biological properties affect water and nutrient availability, which often vary over small distances. This results in different parts of the root system being exposed to distinct soil conditions (Vetterlein et al. 2004; Tavakkoli et al. 2010). Root system architecture (RSA) responds dynamically to these soil properties, which change over time and space. Therefore, RSA phenotypes arise from a combination of plant genetics and soil conditions (Rogers and Benfey 2015; Khan et al. 2016; Correa et al. 2019). The increased root/shoot ratio observed in soil-cultivated plants is advantageous for plants mining water and nutrients, a phenomenon previously reported in wild and modern barley lines (Suresh et al. 2024).

On the other hand, hydroponic cultures use aqueous media that provide a more homogeneous environment with a high nutrient concentration. In addition, hydroponics lack mechanical limitations for root growth, and the entire nutrient mixture is readily available to plants. As a result, excessive investment in root growth is unnecessary, allowing plants to allocate more biomass to above-ground organs (Asher and Edwards 1983; Sardare and Admane 2013; Sharma et al. 2018). This finding is consistent with the observed increase in shoot dry weight across all six species (Fig. S1b). Furthermore, leafy vegetables such as spinach have been shown to yield higher yields when cultivated hydroponically (Ranawade et al. 2017).

### Histochemical detection of suberin and lignin

Histochemical analysis revealed a significantly greater degree of suberization (Figs. 2A and 3A) and lignification (Figs. 2B and 3B) in the soil-grown roots than in the hydroponically cultivated roots across all 6 species. Since both suberin and lignin deposition are classical plant responses to environmental stress, these results clearly indicate that soil, as a cultivation medium, imposes greater stress on roots than does hydroponics. This includes potential mechanical restrictions and uneven distributions of water and nutrients, which, as mentioned earlier, are not issues in aqueous cultivation media.

In contrast to barley, the endodermis of Zone A in soil-cultivated wheat roots was nearly fully suberized (Fig. 2Ap), whereas suberization in hydroponically cultivated roots only began at approximately 25% of the root length (Fig. 2Ak). This observation aligns with previous studies that reported the initiation of endodermal suberization in soil-cultivated wheat roots starting at 30 to 40 mm above the root apex (Ouyang et al. 2020), which is roughly equivalent to Zone A in the present study. For corn, the observation of the endodermal suberin lamellae of soil-grown roots at similar root lengths has also been previously reported for both cultivation conditions (Zimmermann et al. 2000; Abiko et al. 2012). However, in contrast to these studies, where exodermal formation was reportedly absent in hydroponically grown plants (Zimmermann et al. 2000; Schreiber et al. 2005), we detected exodermal suberization at 50% of the root length in hydroponically grown roots.

In tomatoes, the root suberization observed in hydroponics was consistent with findings for plants grown in MS media. Interestingly, unlike other species, tomatoes rely almost exclusively on exodermal suberin to regulate nutrient uptake in the root (Kajala et al. 2021; Cantó-Pastor et al. 2024). The deposition of suberin lamellae in the roots of broad bean in hydroponic solutions was consistent with previous reports (Calvo-Polanco et al. 2014).

For all soil-grown monocotyledonous species and the exodermis of corn, the endodermis presented distinct U-shaped lignin deposition starting at 50% of the root length, which is characteristic of the tertiary stage of endodermal development (Geldner 2013; Shen et al. 2015). This structure was observed only in hydroponically grown monocotyledonous plants at 90% root length and was absent in dicotyledonous species.

### Chemical analysis of root suberin and lignin contents

The results from the chemical analysis (Figs. 4 and 5) confirmed the observations made through microscopy (Figs. 2 and 3), with the total suberin and lignin contents in soil-cultivated roots consistently being significantly greater than those grown in hydroponics. Aromatic suberin contents (Fig. S3a) followed the same trend, but the data revealed considerable variability among replicates for each species, and the aromatic suberin content was greater than the aliphatic suberin content. Since aromatic monomers are not specific to suberin and can also originate from primary cell walls, this variability could lead to an overestimation of aromatic suberin levels (Chabbert et al. 1994; Carpita 1996; Ranathunge et al. 2016). The most abundant aliphatic suberin monomers were ω-OH acids and diacids (Fig. S2), a pattern that has been reported in other studies (Schreiber et al. 2005; Ranathunge et al. 2011; Kreszies et al. 2019). These monomers are thought to be abundant due to their structural properties, as they have two different functional groups (OH and COOH) at either end of the hydrocarbon chain, enabling them to form a three-dimensional suberin structure. In contrast, monofunctional fatty acids and alcohols, which have a single functional group, serve as dead ends in suberin macromolecules (Graça 2015; Ranathunge et al. 2016).

Among the six species studied, G-lignin was the most abundant lignin subunit, followed by S-lignin. When dicotyledonous and monocotyledonous species are compared, dicotyledonous species present higher G- and S-lignin values than monocotyledonous species do, which is consistent with previously reported findings on lignin composition in different plant species (Boerjan et al. 2003; Liu et al. 2018).

In hydroponic studies, apoplastic barrier formation is influenced by various environmental stimuli, some of which induce it, whereas others delay it (Grünhofer et al. 2021). Osmotic stress, for example, has been shown to increase suberization in barley, corn, and wheat (Zimmermann et al. 2000; Shen et al. 2015; Kreszies et al. 2019; Terletskaya et al. 2020). However, in the present experiment, the two cultivation conditions were essentially stress-free control conditions: the soil had a water potential of – 0.06 ± 0.03 MPa, whereas the nutrient solution had less than – 0.02 MPa when measured. The pronounced increase in apoplastic barrier formation in soil-grown roots may be attributed to differences in nutrient availability. Specifically, the concentrations of K_2_O and Mg differed between the two conditions. K_2_O was present at 201 mg/l in soil, whereas in hydroponics, it was present at 117 mg/l, and Mg was present at 112 mg/l in soil compared with 24 mg/l in hydroponics (Supplementary Table S5). In hydroponic systems, nutrient gradients do not develop, and neither nutrient depletion nor nutrient accumulation in the rhizosphere occurs, unlike in soil-grown plants, where these processes are common for several elements (Barber and Ozanne 1970; Tavakkoli et al. 2010; Kuzyakov and Razavi 2019).

In addition, water in soil is attracted by the solid matrix, and nutrients with charges are bound to the positive and negative charges of soil particles (Iwata et al. 1996; Kuzyakov and Razavi 2019). Therefore, ion and water availability and, consequently, the water potential of the soil in the rhizosphere around roots growing in soil can vary significantly. In sharp contrast, hydroponic systems maintain a matric potential near zero (Tavakkoli et al. 2010; Kuzyakov and Razavi 2019), leading to more uniform conditions for plant roots.

### Differential gene expression analyses between soil-grown and hydroponically grown roots of barley

A comparison of SZA vs. HZA revealed that the top GO terms for the upregulated genes included ribosomal processes and, hence, protein machinery and mitochondrial electron transport chain components (Supplementary Table S2), suggesting that these gene categories are more highly expressed in Zone A in soil than in hydroponics. Ribosomal processes, along with components of the mitochondrial electron transport chain, are linked to crucial stress signaling pathways in plants (Dourmap et al. 2020; Fakih et al. 2023), supporting the idea that roots in soil face more challenging environmental conditions than those in hydroponics. However, the specific roles of these genes in apoplastic suberization or lignification remain to be explored. The top 100 upregulated genes (Fig. S6, Supplementary Table S6) also yielded GO terms related to ribosome machinery and protein synthesis, reinforcing the importance of translation processes in soil-grown roots.

In the comparison of SZA vs. HZB, the upregulated genes were associated primarily with primary metabolic processes such as acetyl-CoA metabolic processes, glutathione metabolic processes, and pyruvate metabolic processes. These processes have also not been directly associated with suberin- and lignin-mediated signaling in plants. The top 100 upregulated genes in this category yielded terms similar to those related to ribosomal machinery and protein synthesis, akin to those observed in the SZA vs. HZA comparison. Furthermore, commonly upregulated genes between these comparisons were associated with ribosomal processes, highlighting the transcriptional importance of the translation machinery when plants are grown in two contrasting growth media.

We also identified downregulated genes in our transcriptomic datasets when we compared SZA with HZA and HZB. The top GO terms for the downregulated genes in SZA vs. HZA were similar to those enriched in SZA vs. HZB, including acetyl-CoA metabolic process, glutathione metabolic process, and cell wall organization. These findings suggest a similarity in the nature of genes in the two different zones of hydroponically grown roots. Moreover, downregulated genes in SZA vs. HZB were largely associated with the biotic stress response and signaling in plants, such as defense responses to bacteria and the cell surface receptor signaling pathway (Supplementary Table S2). This is an interesting observation, as it represents one of the first reports linking biotic stress responsive genes to possible functions during growth in different cultivation systems, i.e., soil and hydroponics. Given that different zones exhibit varying levels of suberization, potentially a defense mechanism (Chen et al. 2022), this may indicate an interplay between biotic stress components and suberin machinery during growth under these two conditions.

For RNA-Seq, Zones A of soil-grown roots (SZA: partially suberized, 0–12.5%) were compared with hydroponically grown root data from Kreszies et al. (2019) Zone A (HZA: nonsuberized, 0–12.5%) and Zone B (HZB: partially suberized, 25–37.5%). We hypothesized that SZA would regulate genes related to apoplastic barrier formation similarly to HZB. Indeed, genes related to suberization (FAD, KCS, and CYP) were significantly upregulated in SZA compared with HZA, whereas these differences mostly disappeared compared with those in HZB (Fig. 7). Genes associated with the phenylpropanoid pathway, also involved in lignification (PAL, HCT, FAH, OMT), were strongly upregulated in SZA compared with both HZA and HZB, whereas peroxidases (PRX and PER) related to lignification were mostly upregulated in SZA compared with HZB. KEGG analyses of the DEGs confirmed that the upregulated genes are involved in various steps of lignin biosynthesis (Fig. S7), including PER39, PRX52, and PRX72, which have been shown to be involved in lignin biosynthesis, particularly in Casparian strip lignification (Herrero et al. 2013; Fernández-Pérez et al. 2015a, 2015b; Hoffmann et al. 2020; Rojas-Murcia et al. 2020).

Overall, genes related to suberin and lignin biosynthesis were significantly more highly upregulated in the roots of soil-grown plants than in those of hydroponically grown plants, indicating that, compared with hydroponic cultivation, soil provides a more stressful environment for root growth. This enhanced formation of apoplastic barriers in soil-grown roots is likely to shuttle radial water to the symplastic path of water transport. These findings suggest that plasma membrane aquaporins may be upregulated in response to stress (Kreszies et al. 2020). In fact, the expression of aquaporin genes such as NIP1;5, NIP1;2, PIP2;8, PIP2;2, PIP2;1, PIP2;4, TIP1;3, TIP2;2, and TIP2;3 was upregulated in SZA compared with HZA and HZB (Fig. S5, Supplementary Table S4). These findings indicate that soil-grown roots develop apoplastic barriers more rapidly than hydroponically grown roots do. Interestingly, nutrient transporters were more highly upregulated in HZA and HZB than in SZA, despite higher nutrient availability in hydroponics than in soil (Fig. S8, Supplementary Table S7).

## Conclusion

This study revealed that cultivation media have a pronounced effect on plant morphology, root system architecture, root suberization, and lignification. This finding is supported by the observed differential gene expression in barley roots grown in two contrasting cultivation media: soil and hydroponics. The physicochemical characteristics of the cultivation media (soil vs. hydroponics) significantly affect root development, which should be considered when hydroponic cultivation is used.

## Supplementary Information

Below is the link to the electronic supplementary material.

Supplementary file1 (XLSX 5430 KB)

Supplementary file2 (XLSX 2712 KB)

Supplementary file3 (DOCX 88 KB)

Supplementary file4 (DOCX 58 KB)

Supplementary file5 (DOCX 18 KB)

Supplementary file6 (XLSX 49 KB)

Supplementary file7 (DOCX 102 KB)

Supplementary file8 (DOCX 4998 KB)

## Data Availability

Data that support the findings of this study are available from the corresponding author upon reasonable request.

## Abbreviations

DEGs: Differentially expressed genes
HZA: Zone A of hydroponically grown roots
HZB: Zone B of hydroponically grown roots
SZA: Zone A of soil-grown roots
