# Supplementary material for: Comparing apoplastic root barrier formation and morphology in six crop species cultivated in soil vs. hydroponics
Source: Planta. 2025 Nov 1;262(6):141. doi: 10.1007/s00425-025-04862-3 (PMC12579656; doi:10.1007/s00425-025-04862-3)
Supplement: Supplementary file 3 — Supplementary file3 (DOCX 88 KB) [file 425_2025_4862_MOESM3_ESM.docx]

**Supporting Information**

**Table S3:** DEGs up- and down-regulated related to suberin and lignin related genes with references

**Journal Name:** Planta: An International Journal of Plant Biology

**Article title:** Comparing apoplastic root barrier formation and morphology in six crop species cultivated in soil vs. hydroponics

Authors: **Jorge Carvajal^1*#^, Kiran Suresh^1#^, Sabarna Bhattacharyya^2^, Viktoria V. Zeisler-Diehl^1^, Tobias Wojciechowski^3^, Lukas Schreiber^1^**

^1^Department of Ecophysiology, Institute of Cellular and Molecular Botany, University of Bonn, Kirschallee 1, 53115 Bonn, Germany; ^2^Plant Cell Biology, Institute of Cellular and Molecular Botany, University of Bonn, Kirschallee 1, 53115 Bonn, Germany; ^3^Plant Sciences (IBG-2), Forschungszentrum Jülich GmbH, D-52425 Jülich, Germany. ^#^Contributed equally

***Author for correspondence:** Jorge Carvajal

**E-Mail:** jcar@uni-bonn.de

| **At_ID** | **Description** | **Hv_ID** | **I %** | **ZA log2FC** | **ZB log2FC** | **References** |
| --- | --- | --- | --- | --- | --- | --- |
| AT3G12120 | FAD2 \| fatty acid desaturase 2 | HORVU6Hr1G070420 | 68 | 1.13 | 1.30 | Major enzyme responsible for the synthesis of 18:2 **fatty acids** in the endoplasmic reticulum. Na+/H+ exchange activity and abiotic stresses. (Nguyen et al., 2019; Zhang et al., 2012) |
|  |  | HORVU6Hr1G070540 | 60 | 2.53 | 2.26 |  |
| AT5G05580 | FAD8 \| fatty acid desaturase 8 | HORVU4Hr1G024950 | 68 | 1.14 |  | PUFA: Trienoic **fatty acids**, leaf: Acts in autoregulatory fashion to destabilize the protein at high temperature. (Maeda et al., 2008; Matsuda et al., 2005) |
| AT2G26640 | KCS11 | HORVU1Hr1G090730 | 74 | 1.41 |  | Encodes KCS11/12/17, a member of the 3-ketoacyl-CoA synthase family involved in the biosynthesis of **VLCFA**, fatty acid elongase (**FAE**). (Batsale et al., 2023; Joubès et al., 2008) |
| AT2G28630 | KCS12 | HORVU6Hr1G089750 | 57 | 2.33 |  |  |
| AT4G34510 | KCS17 | HORVU7Hr1G042070 | 62 | 4.30 | 4.04 |  |
| AT3G11980 | MS2, FAR2 | HORVU2Hr1G086620 | 57 | 3.72 | 3.45 | Fatty-acyl-CoA reductase (alcohol-forming) activity C16:0 **alcohol.** (Aarts et al., 1997; Doan et al., 2016) |
| AT5G07990 | CYP75B1 | HORVU7Hr1G095900 | 68 | 1.71 |  | CYP75B1 is highly expressed in siliques, flowers and senescent leaves, in agreement with the distribution of 3’,4’-hydroxylated **flavonoids.** (Schoenbohm et al., 2000) |
| AT2G27690 | CYP94C1 | HORVU3Hr1G084450 | 57 | 1.25 |  | Non-A P450s related to fatty acid and alkane oxidizing enzymes, Involvement in fatty acid **ω-hydroxylation**; the biosynthesis of cutin monomers or defense reactions. (Tijet et al., 1998) |
| AT3G56630 | CYP94D2 | HORVU3Hr1G108150 | 44 | 3.72 |  |  |
| AT1G01610 | GPAT4 | HORVU3Hr1G029770 | 56 | 1.37 | -3.30 | **Bifunctional sn-glycerol-3-phosphate 2-O-acyltransferase/phosphatase. Involved in cutin assembly and is functionally redundant with GPAT8.** (Li et al., 2007) |
| AT1G06520 | GPAT1 | HORVU3Hr1G056830 | 59 | 2.16 |  | Tapetum and pollen development, GPAT1 in regulating Arabidopsis plant height, **cell wall organization** and biogenesis. (Bai et al., 2021) |
| AT3G11430 | GPAT5 | HORVU1Hr1G072590 | 82 | 1.41 | -1.60 | **Sn-glycerol-3-phosphate 2-O-acyltransferas, involved in the biosynthesis of suberin polyester.** (Beisson et al., 2007) |
| AT4G01950 | GPAT3 | HORVU2Hr1G023650 | 55 | 1.29 | -1.66 | Glycerol-3-phosphate to lysophosphatidic acid in the synthesis of **triacylglycerol.** (Bertolesi et al., 2012; Wendel et al., 2009) |
|  |  | HORVU5Hr1G116920 | 47 | 3.28 |  |  |

**DEGs of suberin biosynthesis up-regulated in zone A of soil grow roots when compared to hydroponic roots zones A and B**

**DEGs of phenylpropanoid biosynthesis up-regulated in zone A of soil grow roots when compared to hydroponic roots zones A and B.**

| **At_ID** | **Description** | **Hv_ID** | **I %** | **ZA log2FC** | **ZB log2FC** | **References** |
| --- | --- | --- | --- | --- | --- | --- |
| AT3G53260 | PAL2 | HORVU6Hr1G058820 | 74 | 4.53 | 3.12 | **Encodes phenylalanine lyase;** flavonoid and **lignin** biosynthesis (Cochrane et al., 2004; Rohde et al., 2004) |
| AT5G48930 | HCT | HORVU2Hr1G109430 | 33 | 1.50 |  | **Encode for the hydroxycinnamoyl-Coenzyme A shikimate/quinate hydroxycinnamoyltransferase (HCT) both synthesizing and catabolizing the hydroxycinnamoylesters (coumaroyl/caffeoyl shikimate and quinate) involved in the phenylpropanoid pathway** (Eudes et al., 2016; L. Hoffmann et al., 2004) |
|  |  | HORVU2Hr1G112120 | 49 |  | 1.96 |  |
|  |  | HORVU3Hr1G097120 | 39 | 4.97 | 5.25 |  |
|  |  | HORVU4Hr1G077790 | 27 | 3.96 | 5.15 |  |
|  |  | HORVU7Hr1G030780 | 36 |  | 1.03 |  |
|  |  | HORVU7Hr1G118090 | 36 | 2.88 |  |  |
| AT2G34770 | FAH1 | HORVU5Hr1G100890 | 73 |  | 1.58 | **Encodes a fatty acid hydroxylase, F5H - lignin biosynthesis** (Aguadé, 2001; Nagano et al., 2012**)** |
| AT4G36220 | F5H1, CYP84A1 | HORVU2Hr1G109440 | 65 | 1.64 |  | **ferulic acid 5-hydroxylase 1, Involved in lignin biosynthesis** (Anderson et al., 2015; Weng et al., 2010) |
| AT5G54160 | OMT1, O-methyltransferase | HORVU1Hr1G003370 | 60 | 1.95 | 4.16 | **A caffeic acid/5-hydroxyferulic acid O-methyltransferase lignin biosynthesis;** Aromatic metabolism (Do et al., 2007; Moinuddin et al., 2010; Weng et al., 2010) |
|  |  | HORVU1Hr1G011930 | 57 | 4.67 | 8.51 |  |
|  |  | HORVU4Hr1G085680 | 29 | 9.05 | 1.61 |  |
|  |  | HORVU7Hr1G119480 | 50 |  | 1.10 |  |
| AT2G22420 | PRX17 | HORVU5Hr1G048170 | 78 | 4.26 |  | **Encodes a cell wall-localized class III peroxidase that is directly regulated by the MADS-box transcription factor AGL15 and is involved in lignified tissue formation** (Cosio et al., 2017) |
|  |  | HORVU5Hr1G070290 | 71 | 1.75 |  |  |
| AT2G41480 | ATPRX25, PEROXIDASE 25, PRX25 | HORVU0Hr1G006290 | 65 | 2.80 | 1.83 | **Encodes a cationic cell-wall-bound peroxidase homolog that is involved in the lignification of cell walls** (Renard et al., 2020; Shigeto et al., 2014, 2015) |
|  |  | HORVU3Hr1G024510 | 65 | 4.53 | 3.38 |  |
| AT4G11290 | PER39, PEROXIDASE 39 | HORVU0Hr1G002800 | 45 | -2.35 | 2.69 | **Peroxidase required for Casparian strip lignification as well as partially required for SGN-dependent compensatory lignification.**(Rojas-Murcia et al., 2020) |
|  |  | HORVU1Hr1G016770 | 36 |  | 2.05 |  |
|  |  | HORVU1Hr1G069000 | 48 |  | 1.66 |  |
|  |  | HORVU2Hr1G026380 | 48 |  | 4.81 |  |
|  |  | HORVU2Hr1G026520 | 42 |  | 4.01 |  |
|  |  | HORVU2Hr1G026540 | 43 |  | 3.82 |  |
|  |  | HORVU2Hr1G026590 | 43 |  | 2.36 |  |
|  |  | HORVU7Hr1G010280 | 42 |  | 4.20 |  |
| AT5G05340 | PEROXIDASE 52, PRX52 | HORVU2Hr1G018430 | 63 | 5.86 | 6.23 | Prx52 is involved in the synthesis of S units in interfascicular fibers at late stages of the **lignification** process (Fernández-Pérez et al., 2015b) |
|  |  | HORVU2Hr1G018520 | 61 |  | 1.65 |  |
|  |  | HORVU2Hr1G024280 | 60 | 4.28 | 4.89 |  |
|  |  | HORVU3Hr1G026010 | 56 |  | 3.49 |  |
|  |  | HORVU4Hr1G052060 | 62 |  | 2.22 |  |
|  |  | HORVU6Hr1G026600 | 66 | 1.07 | 1.53 |  |
|  |  | HORVU7Hr1G089300 | 65 | 1.95 | 4.72 |  |
|  |  | HORVU7Hr1G089320 | 59 |  | 3.22 |  |
|  |  | HORVU7Hr1G089360 | 60 | 1.75 |  |  |
|  |  | HORVU7Hr1G089370 | 60 |  | 2.43 |  |
| AT5G42180 | PER64, PEROXIDASE 64, PRX64 | HORVU0Hr1G010070 | 38 | 1.09 | 1.44 | **Peroxidase required for Casparian strip lignification as well as partially required for SGN-dependent compensatory lignification** (Rojas-Murcia et al., 2020; Tokunaga et al., 2009; Yi Chou et al., 2018) |
| AT5G66390 | PER72, PEROXIDASE 72, PRX72 | HORVU3Hr1G036820 | 69 | 1.53 | 3.29 | **Encodes a peroxidase that is involved in lignin biosynthesis. Required for Casparian strip lignification as well as partially required for SGN-dependent compensatory lignification** (Fernández-Pérez et al., 2015a; Herrero et al., 2013; Hoffmann et al., 2020; Rojas-Murcia et al., 2020) |
|  |  | HORVU3Hr1G036860 | 67 |  | 1.80 |  |
|  |  | HORVU3Hr1G036880 | 75 |  | 2.64 |  |
|  |  | HORVU6Hr1G075510 | 74 | 1.65 | 2.94 |  |
|  |  | HORVU7Hr1G083550 | 44 | 2.09 |  |  |
| AT1G28680 | HXXXD-type acyl-transferase family protein | HORVU0Hr1G019500 | 50 | 1.78 |  | **Catalyses trans-cis isomerization and lactonization in the biosynthesis of coumarins in roots.**(Vanholme et al., 2019) |
| AT1G31490 |  | HORVU7Hr1G021430 | 31 |  | 7.37 |  |
|  |  | HORVU7Hr1G022010 | 31 |  | 2.62 |  |
|  |  | HORVU7Hr1G022080 | 31 | 4.32 | 3.56 |  |
| AT1G32910 |  | HORVU2Hr1G100150 | 56 | 1.56 | 2.69 |  |
| AT2G39980 |  | HORVU3Hr1G085100 | 53 | 2.37 |  | Microarray analyses of cross-talk between jasmonic acid and ethylene signaling, **Root** **cell** **elongation** (Markakis et al., 2012; Zimmermann et al., 2004) |
| AT3G29670 |  | HORVU7Hr1G030730 | 32 | 1.20 | 1.59 | **Encodes a malonyltransferase that may play a role in phenolic xenobiotic detoxification. The mRNA is cell-to-cell mobile. PMAT2** |
| AT3G62160 |  | HORVU1Hr1G017410 | 40 | 1.70 |  |  |
|  |  | HORVU1Hr1G026520 | 42 | 1.85 | 2.13 |  |
|  |  | HORVU2Hr1G000410 | 31 | 1.49 |  |  |
|  |  | HORVU3Hr1G020230 | 39 |  | 1.16 |  |
|  |  | HORVU3Hr1G022340 | 41 | 2.93 |  |  |
|  |  | HORVU3Hr1G066740 | 41 | 1.79 | 2.67 |  |
|  |  | HORVU3Hr1G096360 | 37 | 1.75 | -6.25 |  |
|  |  | HORVU4Hr1G087510 | 32 | 2.28 |  |  |
| AT4G13840 |  | HORVU2Hr1G099550 | 34 | 1.79 | 1.51 | TAIR Aliases: **CER26** Clade of the **BAHD** Acyltransferase **;** very long chain fatty acid elongation (Haslam et al., 2017; Pascal et al., 2013) |
| AT5G17540 |  | HORVU4Hr1G080100 | 42 | 8.51 | 7.99 | Fructokinase-like; Biosynthesis of cellulose (Andersson-Gunnerås et al., 2006) |
| AT5G39090 |  | HORVU4Hr1G072840 | 32 |  | 1.33 | Anthocyanin 5-aromatic acyltransferase, BAHD acyl transferases (Buchanan-Wollaston et al., 2005; Luo et al., 2007) |
| AT5G41040 |  | HORVU2Hr1G035810 | 64 | 3.15 | 1.20 | TAIR Aliases: **ASFT, HHT, RWP1 suberin** aromatics (Gou et al., 2009; Serra et al., 2010) |
|  |  | HORVU2Hr1G035830 | 64 | 1.61 |  |  |
| AT5G67150 |  | HORVU2Hr1G006540 | 35 | 2.92 | 4.66 |  |
|  |  | HORVU5Hr1G118600 | 37 | 1.49 |  |  |
| AT2G38080 | IRX12, LAC4, LMCO4 | HORVU4Hr1G027050 | 67 | 2.02 | -3.62 | LAC4 is expressed in vascular bundles and fibers and likely contributes to **lignin** biosynthesis, and hence cell wall biosynthesis, there. lac4/irx12 mutants have a mild irregular xylem phenotype. (Berthet et al., 2011; Brown et al., 2005; Yi Chou et al., 2018) |
| AT3G09220 | LAC7 | HORVU2Hr1G002440 | 57 | 5.85 | 4.27 | **Lignin**, putative laccase, a member of laccase family of genes (17 members in Arabidopsis).(Wei et al., 2018; Yi Chou et al., 2018) |
|  |  | HORVU3Hr1G085520 | 63 | 2.38 | 1.86 |  |
|  |  | HORVU1Hr1G072470 | 70 |  | 2.03 |  |
|  |  | HORVU1Hr1G072530 | 63 | 1.95 |  |  |
|  |  | HORVU2Hr1G041290 | 72 |  | 1.81 |  |
|  |  | HORVU6Hr1G013870 | 64 | 1.74 |  |  |

**References:**

- Aarts, M. G., Hodge, R., Kalantidis, K., Florack, D., Wilson, Z. A., Mulligan, B. J., Stiekema, W. J., Scott, R., & Pereira, A. (1997). The Arabidopsis MALE STERILITY 2 protein shares similarity with reductases in elongation/condensation complexes. *The Plant Journal: For Cell and Molecular Biology*, *12*(3), 615–623. https://doi.org/10.1046/j.1365-313x.1997.00615.x
- Aguadé, M. (2001). Nucleotide Sequence Variation at Two Genes of the Phenylpropanoid Pathway, the FAH1 and F3H Genes, in Arabidopsis thaliana. *Molecular Biology and Evolution*, *18*(1), 1–9. https://doi.org/10.1093/oxfordjournals.molbev.a003714
- Anderson, N. A., Bonawitz, N. D., Nyffeler, K., & Chapple, C. (2015). Loss of FERULATE 5-HYDROXYLASE Leads to Mediator-Dependent Inhibition of Soluble Phenylpropanoid Biosynthesis in Arabidopsis. *Plant Physiology*, *169*(3), 1557–1567. https://doi.org/10.1104/pp.15.00294
- Andersson-Gunnerås, S., Mellerowicz, E. J., Love, J., Segerman, B., Ohmiya, Y., Coutinho, P. M., Nilsson, P., Henrissat, B., Moritz, T., & Sundberg, B. (2006). Biosynthesis of cellulose-enriched tension wood in Populus: Global analysis of transcripts and metabolites identifies biochemical and developmental regulators in secondary wall biosynthesis. *The Plant Journal*, *45*(2), 144–165. https://doi.org/10.1111/j.1365-313X.2005.02584.x
- Bai, Y., Shen, Y., Zhang, Z., Jia, Q., Xu, M., Zhang, T., Fang, H., Yu, X., Li, L., Liu, D., Qi, X., Chen, Z., Wu, S., Zhang, Q., & Liang, C. (2021). A GPAT1 Mutation in Arabidopsis Enhances Plant Height but Impairs Seed Oil Biosynthesis. *International Journal of Molecular Sciences*, *22*(2), Article 2. https://doi.org/10.3390/ijms22020785
- Batsale, M., Alonso, M., Pascal, S., Thoraval, D., Haslam, R. P., Beaudoin, F., Domergue, F., & Joubès, J. (2023). Tackling functional redundancy of Arabidopsis fatty acid elongase complexes. *Frontiers in Plant Science*, *14*, 1107333. https://doi.org/10.3389/fpls.2023.1107333
- Beisson, F., Li, Y., Bonaventure, G., Pollard, M., & Ohlrogge, J. B. (2007). The acyltransferase GPAT5 is required for the synthesis of suberin in seed coat and root of Arabidopsis. *The Plant Cell*, *19*(1), 351–368. https://doi.org/10.1105/tpc.106.048033
- Berthet, S., Demont-Caulet, N., Pollet, B., Bidzinski, P., Cézard, L., Le Bris, P., Borrega, N., Hervé, J., Blondet, E., Balzergue, S., Lapierre, C., & Jouanin, L. (2011). Disruption of LACCASE4 and 17 Results in Tissue-Specific Alterations to Lignification of Arabidopsis thaliana Stems. *The Plant Cell*, *23*(3), 1124–1137. https://doi.org/10.1105/tpc.110.082792
- Bertolesi, G. E., Iannattone, S., Johnston, J., Zaremberg, V., & McFarlane, S. (2012). Identification and expression analysis of GPAT family genes during early development of Xenopus laevis. *Gene Expression Patterns*, *12*(7), 219–227. https://doi.org/10.1016/j.gep.2012.04.002
- Brown, D. M., Zeef, L. A. H., Ellis, J., Goodacre, R., & Turner, S. R. (2005). Identification of novel genes in Arabidopsis involved in secondary cell wall formation using expression profiling and reverse genetics. *The Plant Cell*, *17*(8), 2281–2295. https://doi.org/10.1105/tpc.105.031542
- Buchanan-Wollaston, V., Page, T., Harrison, E., Breeze, E., Lim, P. O., Nam, H. G., Lin, J.-F., Wu, S.-H., Swidzinski, J., Ishizaki, K., & Leaver, C. J. (2005). Comparative transcriptome analysis reveals significant differences in gene expression and signalling pathways between developmental and dark/starvation-induced senescence in Arabidopsis. *The Plant Journal*, *42*(4), 567–585. https://doi.org/10.1111/j.1365-313X.2005.02399.x
- Cochrane, F. C., Davin, L. B., & Lewis, N. G. (2004). The Arabidopsis phenylalanine ammonia lyase gene family: Kinetic characterization of the four PAL isoforms. *Proteomics 1*, *65*(11), 1557–1564. https://doi.org/10.1016/j.phytochem.2004.05.006
- Cosio, C., Ranocha, P., Francoz, E., Burlat, V., Zheng, Y., Perry, S. E., Ripoll, J.-J., Yanofsky, M., & Dunand, C. (2017). The class III peroxidase PRX17 is a direct target of the MADS-box transcription factor AGAMOUS-LIKE15 (AGL15) and participates in lignified tissue formation. *The New Phytologist*, *213*(1), 250–263. https://doi.org/10.1111/nph.14127
- Do, C.-T., Pollet, B., Thévenin, J., Sibout, R., Denoue, D., Barrière, Y., Lapierre, C., & Jouanin, L. (2007). Both caffeoyl Coenzyme A 3-O-methyltransferase 1 and caffeic acid O-methyltransferase 1 are involved in redundant functions for lignin, flavonoids and sinapoyl malate biosynthesis in Arabidopsis. *Planta*, *226*(5), 1117–1129. https://doi.org/10.1007/s00425-007-0558-3
- Doan, T. T. P., Carlsson, A. S., Stymne, S., & Hofvander, P. (2016). Biochemical characteristics of AtFAR2, a fatty acid reductase from Arabidopsis thaliana that reduces fatty acyl-CoA and -ACP substrates into fatty alcohols. *Acta Biochimica Polonica*, *63*(3), 565–570. https://doi.org/10.18388/abp.2016_1245
- Eudes, A., Pereira, J. H., Yogiswara, S., Wang, G., Teixeira Benites, V., Baidoo, E. E. K., Lee, T. S., Adams, P. D., Keasling, J. D., & Loqué, D. (2016). Exploiting the Substrate Promiscuity of Hydroxycinnamoyl-CoA:Shikimate Hydroxycinnamoyl Transferase to Reduce Lignin. *Plant & Cell Physiology*, *57*(3), 568–579. https://doi.org/10.1093/pcp/pcw016
- Fernández-Pérez, F., Pomar, F., Pedreño, M. A., & Novo-Uzal, E. (2015a). Suppression of Arabidopsis peroxidase 72 alters cell wall and phenylpropanoid metabolism. *Plant Science: An International Journal of Experimental Plant Biology*, *239*, 192–199. https://doi.org/10.1016/j.plantsci.2015.08.001
- Fernández-Pérez, F., Pomar, F., Pedreño, M. A., & Novo-Uzal, E. (2015b). The suppression of AtPrx52 affects fibers but not xylem lignification in Arabidopsis by altering the proportion of syringyl units. *Physiologia Plantarum*, *154*(3), 395–406. https://doi.org/10.1111/ppl.12310
- Gou, J.-Y., Yu, X.-H., & Liu, C.-J. (2009). A hydroxycinnamoyltransferase responsible for synthesizing suberin aromatics in Arabidopsis. *Proceedings of the National Academy of Sciences of the United States of America*, *106*(44), 18855–18860. https://doi.org/10.1073/pnas.0905555106
- Haslam, T. M., Gerelle, W. K., Graham, S. W., & Kunst, L. (2017). The Unique Role of the ECERIFERUM2-LIKE Clade of the BAHD Acyltransferase Superfamily in Cuticular Wax Metabolism. *Plants (Basel, Switzerland)*, *6*(2), 23. https://doi.org/10.3390/plants6020023
- Herrero, J., Fernández-Pérez, F., Yebra, T., Novo-Uzal, E., Pomar, F., Pedreño, M. Á., Cuello, J., Guéra, A., Esteban-Carrasco, A., & Zapata, J. M. (2013). Bioinformatic and functional characterization of the basic peroxidase 72 from Arabidopsis thaliana involved in lignin biosynthesis. *Planta*, *237*(6), 1599–1612. https://doi.org/10.1007/s00425-013-1865-5
- Hoffmann, L., Besseau, S., Geoffroy, P., Ritzenthaler, C., Meyer, D., Lapierre, C., Pollet, B., & Legrand, M. (2004). Silencing of hydroxycinnamoyl-coenzyme A shikimate/quinate hydroxycinnamoyltransferase affects phenylpropanoid biosynthesis. *The Plant Cell*, *16*(6), 1446–1465. https://doi.org/10.1105/tpc.020297
- Hoffmann, N., Benske, A., Betz, H., Schuetz, M., & Samuels, A. L. (2020). Laccases and Peroxidases Co-Localize in Lignified Secondary Cell Walls throughout Stem Development1[OPEN]. *Plant Physiology*, *184*(2), 806–822. https://doi.org/10.1104/pp.20.00473
- Joubès, J., Raffaele, S., Bourdenx, B., Garcia, C., Laroche-Traineau, J., Moreau, P., Domergue, F., & Lessire, R. (2008). The VLCFA elongase gene family in Arabidopsis thaliana: Phylogenetic analysis, 3D modelling and expression profiling. *Plant Molecular Biology*, *67*(5), 547–566. https://doi.org/10.1007/s11103-008-9339-z
- Li, Y., Beisson, F., Koo, A. J. K., Molina, I., Pollard, M., & Ohlrogge, J. (2007). Identification of acyltransferases required for cutin biosynthesis and production of cutin with suberin-like monomers. *Proceedings of the National Academy of Sciences*, *104*(46), 18339–18344. https://doi.org/10.1073/pnas.0706984104
- Luo, J., Nishiyama, Y., Fuell, C., Taguchi, G., Elliott, K., Hill, L., Tanaka, Y., Kitayama, M., Yamazaki, M., Bailey, P., Parr, A., Michael, A. J., Saito, K., & Martin, C. (2007). Convergent evolution in the BAHD family of acyl transferases: Identification and characterization of anthocyanin acyl transferases from Arabidopsis thaliana. *The Plant Journal*, *50*(4), 678–695. https://doi.org/10.1111/j.1365-313X.2007.03079.x
- Maeda, H., Sage, T. L., Isaac, G., Welti, R., & DellaPenna, D. (2008). Tocopherols Modulate Extraplastidic Polyunsaturated Fatty Acid Metabolism in Arabidopsis at Low Temperature. *The Plant Cell*, *20*(2), 452–470. https://doi.org/10.1105/tpc.107.054718
- Markakis, M. N., De Cnodder, T., Lewandowski, M., Simon, D., Boron, A., Balcerowicz, D., Doubbo, T., Taconnat, L., Renou, J.-P., Höfte, H., Verbelen, J.-P., & Vissenberg, K. (2012). Identification of genes involved in the ACC-mediated control of root cell elongation in Arabidopsis thaliana. *BMC Plant Biology*, *12*(1), 208. https://doi.org/10.1186/1471-2229-12-208
- Matsuda, O., Sakamoto, H., Hashimoto, T., & Iba, K. (2005). A Temperature-sensitive Mechanism That Regulates Post-translational Stability of a Plastidial ω-3 Fatty Acid Desaturase (FAD8) in Arabidopsis Leaf Tissues. *Journal of Biological Chemistry*, *280*(5), 3597–3604. https://doi.org/10.1074/jbc.M407226200
- Moinuddin, S. G. A., Jourdes, M., Laskar, D. D., Ki, C., Cardenas, C. L., Kim, K.-W., Zhang, D., Davin, L. B., & Lewis, N. G. (2010). Insights into lignin primary structure and deconstruction from Arabidopsis thaliana COMT (caffeic acid O-methyl transferase) mutant Atomt1. *Organic & Biomolecular Chemistry*, *8*(17), 3928–3946. https://doi.org/10.1039/c004817h
- Nagano, M., Takahara, K., Fujimoto, M., Tsutsumi, N., Uchimiya, H., & Kawai-Yamada, M. (2012). Arabidopsis Sphingolipid Fatty Acid 2-Hydroxylases (AtFAH1 and AtFAH2) Are Functionally Differentiated in Fatty Acid 2-Hydroxylation and Stress Responses1[OA]. *Plant Physiology*, *159*(3), 1138–1148. https://doi.org/10.1104/pp.112.199547
- Nguyen, V. C., Nakamura, Y., & Kanehara, K. (2019). Membrane lipid polyunsaturation mediated by FATTY ACID DESATURASE 2 (FAD2) is involved in endoplasmic reticulum stress tolerance in Arabidopsis thaliana. *The Plant Journal: For Cell and Molecular Biology*, *99*(3), 478–493. https://doi.org/10.1111/tpj.14338
- Pascal, S., Bernard, A., Sorel, M., Pervent, M., Vile, D., Haslam, R. P., Napier, J. A., Lessire, R., Domergue, F., & Joubès, J. (2013). The Arabidopsis cer26 mutant, like the cer2 mutant, is specifically affected in the very long chain fatty acid elongation process. *The Plant Journal: For Cell and Molecular Biology*, *73*(5), 733–746. https://doi.org/10.1111/tpj.12060
- Renard, J., Martínez-Almonacid, I., Sonntag, A., Molina, I., Moya-Cuevas, J., Bissoli, G., Muñoz-Bertomeu, J., Faus, I., Niñoles, R., Shigeto, J., Tsutsumi, Y., Gadea, J., Serrano, R., & Bueso, E. (2020). PRX2 and PRX25, peroxidases regulated by COG1, are involved in seed longevity in Arabidopsis. *Plant, Cell & Environment*, *43*(2), 315–326. https://doi.org/10.1111/pce.13656
- Rohde, A., Morreel, K., Ralph, J., Goeminne, G., Hostyn, V., De Rycke, R., Kushnir, S., Van Doorsselaere, J., Joseleau, J.-P., Vuylsteke, M., Van Driessche, G., Van Beeumen, J., Messens, E., & Boerjan, W. (2004). Molecular phenotyping of the pal1 and pal2 mutants of Arabidopsis thaliana reveals far-reaching consequences on phenylpropanoid, amino acid, and carbohydrate metabolism. *The Plant Cell*, *16*(10), 2749–2771. https://doi.org/10.1105/tpc.104.023705
- Rojas-Murcia, N., Hématy, K., Lee, Y., Emonet, A., Ursache, R., Fujita, S., De Bellis, D., & Geldner, N. (2020). High-order mutants reveal an essential requirement for peroxidases but not laccases in Casparian strip lignification. *Proceedings of the National Academy of Sciences of the United States of America*, *117*(46), 29166–29177. https://doi.org/10.1073/pnas.2012728117
- Schoenbohm, C., Martens, S., Eder, C., Forkmann, G., & Weisshaar, B. (2000). Identification of the Arabidopsis thaliana flavonoid 3’-hydroxylase gene and functional expression of the encoded P450 enzyme. *Biological Chemistry*, *381*(8), 749–753. https://doi.org/10.1515/BC.2000.095
- Serra, O., Hohn, C., Franke, R., Prat, S., Molinas, M., & Figueras, M. (2010). A feruloyl transferase involved in the biosynthesis of suberin and suberin-associated wax is required for maturation and sealing properties of potato periderm. *The Plant Journal: For Cell and Molecular Biology*, *62*(2), 277–290. https://doi.org/10.1111/j.1365-313X.2010.04144.x
- Shigeto, J., Itoh, Y., Hirao, S., Ohira, K., Fujita, K., & Tsutsumi, Y. (2015). Simultaneously disrupting AtPrx2, AtPrx25 and AtPrx71 alters lignin content and structure in Arabidopsis stem. *Journal of Integrative Plant Biology*, *57*(4), 349–356. https://doi.org/10.1111/jipb.12334
- Shigeto, J., Nagano, M., Fujita, K., & Tsutsumi, Y. (2014). Catalytic profile of Arabidopsis peroxidases, AtPrx-2, 25 and 71, contributing to stem lignification. *PloS One*, *9*(8), e105332. https://doi.org/10.1371/journal.pone.0105332
- Tijet, N., Helvig, C., Pinot, F., Le Bouquin, R., Lesot, A., Durst, F., Salaün, J. P., & Benveniste, I. (1998). Functional expression in yeast and characterization of a clofibrate-inducible plant cytochrome P-450 (CYP94A1) involved in cutin monomers synthesis. *The Biochemical Journal*, *332 ( Pt 2)*(Pt 2), 583–589. https://doi.org/10.1042/bj3320583
- Tokunaga, N., Kaneta, T., Sato, S., & Sato, Y. (2009). Analysis of expression profiles of three peroxidase genes associated with lignification in Arabidopsis thaliana. *Physiologia Plantarum*, *136*(2), 237–249. https://doi.org/10.1111/j.1399-3054.2009.01233.x
- Vanholme, R., Sundin, L., Seetso, K. C., Kim, H., Liu, X., Li, J., De Meester, B., Hoengenaert, L., Goeminne, G., Morreel, K., Haustraete, J., Tsai, H.-H., Schmidt, W., Vanholme, B., Ralph, J., & Boerjan, W. (2019). COSY catalyses trans-cis isomerization and lactonization in the biosynthesis of coumarins. *Nature Plants*, *5*(10), 1066–1075. https://doi.org/10.1038/s41477-019-0510-0
- Wei, H., Zhao, Y., Xie, Y., & Wang, H. (2018). Exploiting SPL genes to improve maize plant architecture tailored for high-density planting. *Journal of Experimental Botany*, *69*(20), 4675–4688. https://doi.org/10.1093/jxb/ery258
- Wendel, A. A., Lewin, T. M., & Coleman, R. A. (2009). Glycerol-3-phosphate acyltransferases: Rate limiting enzymes of triacylglycerol biosynthesis. *Biochimica et Biophysica Acta (BBA) - Molecular and Cell Biology of Lipids*, *1791*(6), 501–506. https://doi.org/10.1016/j.bbalip.2008.10.010
- Weng, J.-K., Mo, H., & Chapple, C. (2010). Over-expression of F5H in COMT-deficient Arabidopsis leads to enrichment of an unusual lignin and disruption of pollen wall formation. *The Plant Journal: For Cell and Molecular Biology*, *64*(6), 898–911. https://doi.org/10.1111/j.1365-313X.2010.04391.x
- Yi Chou, E., Schuetz, M., Hoffmann, N., Watanabe, Y., Sibout, R., & Samuels, A. L. (2018). Distribution, mobility, and anchoring of lignin-related oxidative enzymes in Arabidopsis secondary cell walls. *Journal of Experimental Botany*, *69*(8), 1849–1859. https://doi.org/10.1093/jxb/ery067
- Zhang, J., Liu, H., Sun, J., Li, B., Zhu, Q., Chen, S., & Zhang, H. (2012). Arabidopsis fatty acid desaturase FAD2 is required for salt tolerance during seed germination and early seedling growth. *PloS One*, *7*(1), e30355. https://doi.org/10.1371/journal.pone.0030355
- Zimmermann, P., Hirsch-Hoffmann, M., Hennig, L., & Gruissem, W. (2004). GENEVESTIGATOR. Arabidopsis microarray database and analysis toolbox. *Plant Physiology*, *136*(1), 2621–2632. https://doi.org/10.1104/pp.104.046367
