# Supplementary material for: Comparing apoplastic root barrier formation and morphology in six crop species cultivated in soil vs. hydroponics
Source: Planta. 2025 Nov 1;262(6):141. doi: 10.1007/s00425-025-04862-3 (PMC12579656; doi:10.1007/s00425-025-04862-3)
Supplement: Supplementary file 4 — Supplementary file4 (DOCX 58 KB) [file 425_2025_4862_MOESM4_ESM.docx]

**Supporting Information**

**Table S4:** DEGs up- and down-regulated related to aquaporin associated genes with references.

**Journal Name:** Planta: An International Journal of Plant Biology

**Article title:** Comparing apoplastic root barrier formation and morphology in six crop species cultivated in soil vs. hydroponics

Authors: **Jorge Carvajal^1*#^, Kiran Suresh^1#^, Sabarna Bhattacharyya^2^, Viktoria V. Zeisler-Diehl^1^, Tobias Wojciechowski^3^, Lukas Schreiber^1^**

^1^Department of Ecophysiology, Institute of Cellular and Molecular Botany, University of Bonn, Kirschallee 1, 53115 Bonn, Germany; ^2^Plant Cell Biology, Institute of Cellular and Molecular Botany, University of Bonn, Kirschallee 1, 53115 Bonn, Germany; ^3^Plant Sciences (IBG-2), Forschungszentrum Jülich GmbH, D-52425 Jülich, Germany. ^#^Contributed equally

***Author for correspondence:** Jorge Carvajal

**E-Mail:** jcar@uni-bonn.de

**DEGs of aquaporin related genes up-regulated in zone A of soil grow roots when compared to hydroponic roots zones A and B.**

| **At_ID** | **Description** | **Hv_ID** | **I %** | **ZA log2FC** | **ZB log2FC** | **References** |
| --- | --- | --- | --- | --- | --- | --- |
| AT4G10380 | NIP5;1, NLM6, NLM8, NOD26-like intrinsic | HORVU1Hr1G047100 | 79 | 1.14 | 2.37 | **Boric acid channel. Required for efficient boron uptake and plant development (**root elongation) **during boron limitation. Functions in arsenate, urea uptake.**(Kato et al., 2009; Yang et al., 2015) |
| AT4G18910 | NIP1;2, NLM2, ATNLM2, NOD26-like intrinsic protein 1;2 | HORVU5Hr1G085710 | 70 | 2.10 | -1.05 | **Encodes an aquaporin homolog. Arsenite, aluminum transport and tolerance. When expressed in yeast cells channels conduct hydrogen peroxide into those cells.** (Y. Wang et al., 2021) |
|  |  | HORVU7Hr1G043590 | 76 | 2.43 |  |  |
|  |  | HORVU7Hr1G043600 | 70 | 1.75 |  |  |
| AT1G01620 | PIP1C, TMP-B, PIP1;3 | HORVU6Hr1G092970 | 89 | 2.28 | 1.78 | **Part of plasma membrane intrinsic protein subfamily PIP1 localizes to the plasma membrane and exhibits water transport activity. Involved redundantly with PIP1;1/2/4/5 in hydraulics and carbon fixation, regulates the expression of related genes that affect plant growth and development. Boron** (Mosa et al., 2016) |
| AT2G16850 | PIP3B, PIP2;8 | HORVU2Hr1G010990 | 81 | 1.17 | 0.79 |  |
|  |  | HORVU2Hr1G089970 | 90 | 3.00 | 2.56 |  |
| AT2G37170 | PIP2B, PIP2;2 | HORVU5Hr1G027240 | 78 | 1.80 | 2.42 | **Plasma membrane intrinsic protein subfamily PIP2, localizes to the plasma membrane and exhibits water transport activity, expressed specifically in the vascular bundles and protein level increases slightly during leaf dev** (Chen et al., 2021; Hernández-Sánchez et al., 2019; Rodrigues et al., 2017; Verdoucq et al., 2008) |
| AT3G53420 | PIP2A, PIP2, PIP2;1 | HORVU6Hr1G058930 | 90 | 1.97 | 2.60 |  |
| AT3G54820 | PIP2D, PIP2;5 | HORVU4Hr1G086900 | 70 | 1.49 |  | **Plasma membrane intrinsic protein 2;5,** increase in their expression levels in **roots** of plants exposed to low air temperature (Jang et al., 2004; Lee et al., 2012) |
| AT4G00430 | PIP1;4, PIP1E, TMP-C, | HORVU5Hr1G055200 | 91 | 2.49 | 1.94 | Permeability of **CO2 and H2O**, effective facilitator of **H2O2**, PIP1;4 and PIP2;5 function in concert to regulate cold acclimation and freezing tolerance responses (Li et al., 2015; Rahman et al., 2020; Tian et al., 2016) |
| AT4G23400 | PIP1D, PIP1;5 | HORVU6Hr1G064140 | 90 | 1.33 |  |  |
| AT5G60660 | PIP2F, PIP2;4 | HORVU2Hr1G038740 | 93 | 1.51 | 1.44 | **A member of the plasma membrane intrinsic protein subfamily PIP2.When expressed in yeast cells can conduct hydrogen peroxide into those cells. Mutants exhibit longer root hairs.**(H. Wang et al., 2020) |
| AT2G25810 | TIP4;1, tonoplast intrinsic protein 4;1 | HORVU3Hr1G031680 | 62 | 3.31 | 3.56 | TIP4;1-YFP are detectable from the **base of the elongation zone**, expressed in **epidermal** and (less strongly) in cortical cells of the differentiation zone(Gattolin et al., 2009) |
| AT4G01470 | GAMMA-TIP3, TIP1;3, ATTIP1.3, tonoplast intrinsic protein 1;3 | HORVU3Hr1G116790 | 78 | 0.82 | 1.03 | **Encodes AtTIP1;3, functions as water and urea channels in pollen** (Soto et al., 2008; Wudick et al., 2014) |
| AT4G17340 | TIP2;2, DELTA-TIP2, tonoplast intrinsic protein 2;2 | HORVU7Hr1G081770 | 93 | 2.50 | 4.70 | **Root expressed tonoplast intrinsic protein.** |
| AT5G47450 | ATTIP2;3, TIP2;3, DELTA-TIP3, tonoplast intrinsic protein 2;3 | HORVU2Hr1G097780 | 76 | 2.42 | 3.97 | **Transports ammonium (NH3) and methylammonium across the tonoplast membrane, gene expression shows diurnal regulation and is upregulated by ammonium** (Loqué et al., 2005) |
|  |  | HORVU6Hr1G062980 | 77 |  | 1.36 |  |

**DEGs of aquaporin related genes down-regulated in zone A of soil grow roots when compared to hydroponic roots zones A and B.**

| **At_ID** | **Description** | **Hv_ID** | **I %** | **ZA log2FC** | **ZB log2FC** | **References** |
| --- | --- | --- | --- | --- | --- | --- |
| AT4G10380 | NIP5;1, NLM6, NLM8, NOD26-like intrinsic | HORVU3Hr1G079560 | 56 | -2.25 |  | **Boric acid channel. Essential for efficient boron uptake and plant development (**root elongation) **under boron limitation. Also functions in arsenate, urea.**(Kato et al., 2009; Yang et al., 2015) |
| AT4G18910 | NIP1;2, NLM2, ATNLM2, NOD26-like intrinsic protein 1;2 | HORVU5Hr1G085710 | 70 | 2.10 | -1.05 | **Encodes an aquaporin homolog. Arsenite, aluminum transport and tolerance. When expressed in yeast cells can conduct hydrogen peroxide into those cells.** (Y. Wang et al., 2021) |
|  |  | HORVU7Hr1G121250 | 70 | -1.13 | -1.15 |  |
| AT5G37820 | NIP4;2, NLM5, NOD26-like intrinsic protein 4;2 | HORVU7Hr1G038220 | 49 | -3.20 | -2.71 | Aquaporins facilitate the transport of **water** and small neutral solutes across cell membranes. |
|  |  | HORVU7Hr1G038270 | 50 | -1.29 | -2.89 |  |
| AT1G01620 | PIP1C, TMP-B, PIP1;3 | HORVU6Hr1G092960 | 90 | -0.68 | -1.19 | **A member of the plasma membrane intrinsic protein subfamily PIP1 localizes to the plasma membrane and exhibits water transport activity Involved redundantly with PIP1;1/2/4/5 in hydraulics and carbon fixation, regulates the expression of related genes that affect plant growth and development. Boron** (Mosa et al., 2016) |
|  |  | HORVU2Hr1G096360 | 95 | -1.20 |  |  |
| AT3G53420 | PIP2A, PIP2, PIP2;1 | HORVU0Hr1G014490 | 74 | -2.16 |  | **A member of the plasma membrane intrinsic protein subfamily PIP2. localizes to the plasma membrane and exhibits water transport activity, expressed specifically in the vascular bundles and protein level increases slightly during leaf dev** (Chen et al., 2021; Hernández-Sánchez et al., 2019; Rodrigues et al., 2017; Verdoucq et al., 2008) |
|  |  | HORVU2Hr1G089820 | 84 | -10.09 | -10.67 |  |
| AT3G54820 | PIP2D, PIP2;5 | HORVU7Hr1G038940 | 69 | -2.22 |  | **Plasma membrane intrinsic protein 2;5,** increase in their expression levels in **roots** of plants exposed to low air temperature (Jang et al., 2004; Lee et al., 2012) |
| AT3G16240 | DELTA-TIP, TIP2;1, DELTA-TIP1, AQP1, ATTIP2;1, delta tonoplast integral protein | HORVU0Hr1G032130 | 82 | -2.41 |  | Aquaporin required to facilitate the transport of **water** from the vacuolar compartment to the cytoplasm, detoxification of ammonium (Loqué et al., 2005) |
| AT3G26520 | TIP2, SITIP, GAMMA-TIP2, TIP1;2, tonoplast intrinsic protein | HORVU2Hr1G013110 | 83 |  | -1.85 | Water channel required to facilitate the transport of **water** across cell membrane. May be involved in the osmoregulation in plants under high osmotic stress such as under a high salt condition (Schüssler et al., 2008) |
| AT3G56950 | SIP2;1, SIP2 small and basic intrinsic protein 2;1 | HORVU4Hr1G052170 | 57 | -1.68 | -2.77 | **One of the Major Intrinsic Proteins (MIPs) which facilitate the passive transport of small molecules across membranes. Belongs to a family of plant aquaporins** (Ishikawa et al., 2005) |

**References:**

- Chen, Q., Liu, R., Wu, Y., Wei, S., Wang, Q., Zheng, Y., Xia, R., Shang, X., Yu, F., Yang, X., Liu, L., Huang, X., Wang, Y., & Xie, Q. (2021). ERAD-related E2 and E3 enzymes modulate the drought response by regulating the stability of PIP2 aquaporins. *The Plant Cell*, *33*(8), 2883–2898. https://doi.org/10.1093/plcell/koab141
- Gattolin, S., Sorieul, M., Hunter, P. R., Khonsari, R. H., & Frigerio, L. (2009). In vivo imaging of the tonoplast intrinsic protein family in Arabidopsis roots. *BMC Plant Biology*, *9*(1), 133. https://doi.org/10.1186/1471-2229-9-133
- Hernández-Sánchez, I. E., Maruri-López, I., Molphe-Balch, E. P., Becerra-Flora, A., Jaimes-Miranda, F., & Jiménez-Bremont, J. F. (2019). Evidence for in vivo interactions between dehydrins and the aquaporin AtPIP2B. *Biochemical and Biophysical Research Communications*, *510*(4), 545–550. https://doi.org/10.1016/j.bbrc.2019.01.095
- Ishikawa, F., Suga, S., Uemura, T., Sato, M. H., & Maeshima, M. (2005). Novel type aquaporin SIPs are mainly localized to the ER membrane and show cell-specific expression in Arabidopsis thaliana. *FEBS Letters*, *579*(25), 5814–5820. https://doi.org/10.1016/j.febslet.2005.09.076
- Jang, J. Y., Kim, D. G., Kim, Y. O., Kim, J. S., & Kang, H. (2004). An expression analysis of a gene family encoding plasma membrane aquaporins in response to abiotic stresses in Arabidopsis thaliana. *Plant Molecular Biology*, *54*(5), 713–725. https://doi.org/10.1023/B:PLAN.0000040900.61345.a6
- Kato, Y., Miwa, K., Takano, J., Wada, M., & Fujiwara, T. (2009). Highly boron deficiency-tolerant plants generated by enhanced expression of NIP5;1, a boric acid channel. *Plant & Cell Physiology*, *50*(1), 58–66. https://doi.org/10.1093/pcp/pcn168
- Lee, S. H., Chung, G. C., Jang, J. Y., Ahn, S. J., & Zwiazek, J. J. (2012). Overexpression of PIP2;5 Aquaporin Alleviates Effects of Low Root Temperature on Cell Hydraulic Conductivity and Growth in Arabidopsis. *Plant Physiology*, *159*(1), 479–488. https://doi.org/10.1104/pp.112.194506
- Li, L., Wang, H., Gago, J., Cui, H., Qian, Z., Kodama, N., Ji, H., Tian, S., Shen, D., Chen, Y., Sun, F., Xia, Z., Ye, Q., Sun, W., Flexas, J., & Dong, H. (2015). Harpin Hpa1 Interacts with Aquaporin PIP1;4 to Promote the Substrate Transport and Photosynthesis in Arabidopsis. *Scientific Reports*, *5*, 17207. https://doi.org/10.1038/srep17207
- Loqué, D., Ludewig, U., Yuan, L., & von Wirén, N. (2005). Tonoplast intrinsic proteins AtTIP2;1 and AtTIP2;3 facilitate NH3 transport into the vacuole. *Plant Physiology*, *137*(2), 671–680. https://doi.org/10.1104/pp.104.051268
- Mosa, K. A., Kumar, K., Chhikara, S., Musante, C., White, J. C., & Dhankher, O. P. (2016). Enhanced Boron Tolerance in Plants Mediated by Bidirectional Transport Through Plasma Membrane Intrinsic Proteins. *Scientific Reports*, *6*, 21640. https://doi.org/10.1038/srep21640
- Rahman, A., Kawamura, Y., Maeshima, M., Rahman, A., & Uemura, M. (2020). Plasma Membrane Aquaporin Members PIPs Act in Concert to Regulate Cold Acclimation and Freezing Tolerance Responses in Arabidopsis thaliana. *Plant & Cell Physiology*, *61*(4), 787–802. https://doi.org/10.1093/pcp/pcaa005
- Rodrigues, O., Reshetnyak, G., Grondin, A., Saijo, Y., Leonhardt, N., Maurel, C., & Verdoucq, L. (2017). Aquaporins facilitate hydrogen peroxide entry into guard cells to mediate ABA- and pathogen-triggered stomatal closure. *Proceedings of the National Academy of Sciences of the United States of America*, *114*(34), 9200–9205. https://doi.org/10.1073/pnas.1704754114
- Schüssler, M. D., Alexandersson, E., Bienert, G. P., Kichey, T., Laursen, K. H., Johanson, U., Kjellbom, P., Schjoerring, J. K., & Jahn, T. P. (2008). The effects of the loss of TIP1;1 and TIP1;2 aquaporins in Arabidopsis thaliana. *The Plant Journal: For Cell and Molecular Biology*, *56*(5), 756–767. https://doi.org/10.1111/j.1365-313X.2008.03632.x
- Soto, G., Alleva, K., Mazzella, M. A., Amodeo, G., & Muschietti, J. P. (2008). AtTIP1;3 and AtTIP5;1, the only highly expressed Arabidopsis pollen-specific aquaporins, transport water and urea. *FEBS Letters*, *582*(29), 4077–4082. https://doi.org/10.1016/j.febslet.2008.11.002
- Tian, S., Wang, X., Li, P., Wang, H., Ji, H., Xie, J., Qiu, Q., Shen, D., & Dong, H. (2016). Plant Aquaporin AtPIP1;4 Links Apoplastic H2O2 Induction to Disease Immunity Pathways. *Plant Physiology*, *171*(3), 1635–1650. https://doi.org/10.1104/pp.15.01237
- Verdoucq, L., Grondin, A., & Maurel, C. (2008). Structure-function analysis of plant aquaporin AtPIP2;1 gating by divalent cations and protons. *The Biochemical Journal*, *415*(3), 409–416. https://doi.org/10.1042/BJ20080275
- Wang, H., Schoebel, S., Schmitz, F., Dong, H., & Hedfalk, K. (2020). Characterization of aquaporin-driven hydrogen peroxide transport. *Biochimica Et Biophysica Acta. Biomembranes*, *1862*(2), 183065. https://doi.org/10.1016/j.bbamem.2019.183065
- Wang, Y., Xiao, E., Wu, G., Bai, Q., Xu, F., Ji, X., Li, C., Li, L., & Liu, J. (2021). The roles of selectivity filters in determining aluminum transport by AtNIP1;2. *Plant Signaling & Behavior*, *16*(12), 1991686. https://doi.org/10.1080/15592324.2021.1991686
- Wudick, M. M., Luu, D.-T., Tournaire-Roux, C., Sakamoto, W., & Maurel, C. (2014). Vegetative and Sperm Cell-Specific Aquaporins of Arabidopsis Highlight the Vacuolar Equipment of Pollen and Contribute to Plant Reproduction1[W]. *Plant Physiology*, *164*(4), 1697–1706. https://doi.org/10.1104/pp.113.228700
- Yang, H., Menz, J., Häussermann, I., Benz, M., Fujiwara, T., & Ludewig, U. (2015). High and Low Affinity Urea Root Uptake: Involvement of NIP5;1. *Plant & Cell Physiology*, *56*(8), 1588–1597. https://doi.org/10.1093/pcp/pcv067
