# Supplementary material for: Comparing apoplastic root barrier formation and morphology in six crop species cultivated in soil vs. hydroponics
Source: Planta. 2025 Nov 1;262(6):141. doi: 10.1007/s00425-025-04862-3 (PMC12579656; doi:10.1007/s00425-025-04862-3)
Supplement: Supplementary file 5 — Supplementary file5 (DOCX 18 KB) [file 425_2025_4862_MOESM5_ESM.docx]

**Supporting Information**

**Table S5** Chemical composition of the two cultivation conditions.

**Journal Name:** Planta: An International Journal of Plant Biology

**Article title:** Comparing apoplastic root barrier formation and morphology in six crop species cultivated in soil vs. hydroponics

Authors: **Jorge Carvajal^1*#^, Kiran Suresh^1#^, Sabarna Bhattacharyya^2^, Viktoria V. Zeisler-Diehl^1^, Tobias Wojciechowski^3^, Lukas Schreiber^1^**

Department of Ecophysiology, Institute of Cellular and Molecular Botany, University of Bonn, Kirschallee 1, 53115 Bonn, Germany; ^2^Plant Cell Biology, Institute of Cellular and Molecular Botany, University of Bonn, Kirschallee 1, 53115 Bonn, Germany; ^3^Plant Sciences (IBG-2), Forschungszentrum Jülich GmbH, D-52425 Jülich, Germany. ^#^Contributed equally

***Author for correspondence:** Jorge Carvajal

E-Mail: [lukas.schreiber@uni-bonn.de](mailto:lukas.schreiber@uni-bonn.de)

| **Nutrient** | **Hydroponics: Half-Strength Hoagland mg/l** | **Soil: PlantaFlor Graberde mg/l** |
| --- | --- | --- |
| Nitrogen | 105 | 122 |
| Ammonium – (NH_4_-N) | 7 | 48 |
| Nitrate – (NO_3_-N) | 98 | 74 |
| Phosphorous (P_2_O_5_) | 15.5 | 18 |
| Potassium (K_2_O) | 117 | 201 |
| Magnesium | 24 | 112 |

**Table S5** Chemical composition of the two cultivation conditions. Soil nutrient data was obtained from the producer (Plantaflor, Vechta), as for half strength Hoagland solution, values were obtained from Jarecki et al., (2005). Concentrations of P and N are similar when comparing the two media, however, there are differences in K_2_O and Mg, with both having a higher concentration in soil compared to hydroponic cultivation
