# Supplementary material for: Comparing apoplastic root barrier formation and morphology in six crop species cultivated in soil vs. hydroponics
Source: Planta. 2025 Nov 1;262(6):141. doi: 10.1007/s00425-025-04862-3 (PMC12579656; doi:10.1007/s00425-025-04862-3)
Supplement: Supplementary file 7 — Supplementary file7 (DOCX 102 KB) [file 425_2025_4862_MOESM7_ESM.docx]

**Supporting Information**

**Table S7:** DEGs up- and down-regulated related to nutrient transporters associated genes with references.

**Journal Name:** Planta: An International Journal of Plant Biology

**Article title:** Comparing apoplastic root barrier formation and morphology in six crop species cultivated in soil vs. hydroponics

Authors: **Jorge Carvajal^1*#^, Kiran Suresh^1#^, Sabarna Bhattacharyya^2^, Viktoria V. Zeisler-Diehl^1^, Tobias Wojciechowski^3^, Lukas Schreiber^1^**

^1^Department of Ecophysiology, Institute of Cellular and Molecular Botany, University of Bonn, Kirschallee 1, 53115 Bonn, Germany; ^2^Plant Cell Biology, Institute of Cellular and Molecular Botany, University of Bonn, Kirschallee 1, 53115 Bonn, Germany; ^3^Plant Sciences (IBG-2), Forschungszentrum Jülich GmbH, D-52425 Jülich, Germany. ^#^Contributed equally

***Author for correspondence:** Jorge Carvajal

E-Mail: jcar@uni-bonn.de

**Table S7:** DEGs up- and down-regulated related to nutrient transporters associated genes with references.

**DEGs of nutrient transporters related genes up-regulated in zone A of soil grow roots when compared to hydroponic roots zones A and B.**

| **At_ID** | **Description** | **Hv_ID** | **I %** | **ZA log2FC** | **ZB log2FC** | **References** |
| --- | --- | --- | --- | --- | --- | --- |
| AT1G08090 | NRT2, NRT2;1, ACH1, LIN1, nitrate transporter 2:1 | HORVU6Hr1G005570 | 68 | 2.33 | 2.80 | **Nitrate transport**, as a dual component transporter with NTR3.1. Acts as a repressor of lateral root initiation (Little et al., 2005; Orsel et al., 2007). |
|  |  | HORVU6Hr1G005580 | 73 | 2.93 | 4.80 |  |
|  |  | HORVU6Hr1G005590 | 73 | 5.30 | 5.57 |  |
| AT1G12110 | NRT1.1, CHL1-1, NRT1, B-1, nitrate transporter 1.1 | HORVU4Hr1G088020 | 60 |  | 5.55 | A dual-affinity **nitrate transporter** found in lateral roots, which promotes lateral root development (Maghiaoui et al., 2020). |
| AT3G45650 | NAXT1, nitrate excretion transporter1 | HORVU7Hr1G025680 | 48 | 2.71 |  | Encodes a **nitrate efflux** transporter NAXT1 (for NITRATE EXCRETION TRANSPORTER1), expressed in the cortex of mature roots (Segonzac et al., 2007). |
| AT5G60770 | NRT2.4, nitrate transporter 2.4 | HORVU6Hr1G005770 | 64 | 4.00 |  | **Member of high affinity nitrate transporter family,** role in both roots and shoots under N starvation (Kiba et al., 2012). |
|  |  | HORVU6Hr1G005930 | 65 | 3.30 |  |  |
| AT4G13510 | AMT1;1, ammonium transporter 1;1 | HORVU2Hr1G088420 | 79 | 1.69 | 1.65 | **Ammonium transporter 1, Participates in concentrative NH4+ acquisition in roots, in long-distance transport to the shoots, and in re-uptake of apoplastic NH4+** (Mayer & Ludewig, 2006)**.** |
|  |  | HORVU6Hr1G057870 | 77 |  | 1.51 |  |
| AT5G43350 | PHT1;1, phosphate transporter 1;1 | HORVU4Hr1G083550 | 73 |  | 1.33 | Phosphate transporter, posttranslational regulation (Bayle et al., 2011; Mudge et al., 2002). |
| AT5G14040 | PHT3;1, phosphate transporter 3;1 | HORVU6Hr1G080480 | 84 |  | 1.04 | Transport of **phosphate groups** from the cytosol to the mitochondrial matrix. Mediates salt stress tolerance through an ATP-dependent pathway and via modulation of the gibberellin metabolism (Zhu et al., 2012) |
| AT1G04690 | KAB1, KV-BETA1, potassium channel beta subunit 1 | HORVU6Hr1G091250 | 85 |  | 1.35 | Probable accessory **potassium channel** protein which modulates the activity of the pore-forming alpha subunit (Tang et al., 1996). |
| AT1G80900 | MGT1, MRS2-10, magnesium transporter 1 | HORVU2Hr1G086410 | 71 | 3.01 | 2.49 | High-affinity **magnesium transporter** that mediates the influx of magnesium (Deng et al., 2006) |
| AT2G01770 | VIT1, ATVIT1, vacuolar iron transporter 1 | HORVU2Hr1G080870 | 62 | 1.33 |  | Encodes an **iron transporter** required for iron sequestration into vacuoles (Kim et al., 2006; Narayanan et al., 2015). |
|  |  | HORVU4Hr1G031840 | 69 | 1.78 | 1.87 |  |
|  |  | HORVU5Hr1G060370 | 62 | 2.69 | 2.77 |  |
| AT2G46800 | ZAT, MTP1, ZAT1, zinc transporter | HORVU2Hr1G082090 | 78 | 1.49 |  | Mediates **zinc** accumulation in roots and confers resistance to zinc (Kawachi et al., 2009; Kobae et al., 2004) |

**DEGs of nutrient transporters related genes down-regulated in zone A of soil grow roots when compared to hydroponic roots zones A and B.**

| **At_ID** | **Description** | **Hv_ID** | **I %** | **ZA log2FC** | **ZB log2FC** | **References** |
| --- | --- | --- | --- | --- | --- | --- |
| AT1G12110 | NRT1.1, CHL1-1, NRT1, B-1, nitrate transporter 1.1 | HORVU7Hr1G071600 | 56 | -1.10 | -1.17 | Dual affinity **nitrate transporter**. Involved in proton-dependent nitrate uptake and in the regulation of the nitrate transporter NRT2.1. Acts also as a nitrate sensor that trigger a specific signaling pathway stimulating lateral root growth and seed germination (Muños et al., 2004; Remans et al., 2006) |
| AT1G32450 | NRT1.5, nitrate transporter 1.5 | HORVU4Hr1G090310 | 52 | -1.55 | -2.10 | **Nitrate transporter** in the transmembrane. a component of the nitrate xylem transfer from the root to the shoot. Lateral root formation (Lin et al., 2008; Zheng et al., 2016). |
|  |  | HORVU6Hr1G070450 | 59 | -2.93 |  |  |
| AT1G69850 | NTL1, NRT1:2, nitrate transporter 1:2 | HORVU2Hr1G085210 | 37 |  | -2.64 | Low-affinity proton-dependent **nitrate transporter**. Involved in constitutive nitrate uptake (Huang et al., 1999). |
|  |  | HORVU2Hr1G085260 | 37 |  | -6.56 |  |
|  |  | HORVU3Hr1G071510 | 41 |  | -1.67 |  |
|  |  | HORVU5Hr1G095020 | 37 |  | -2.72 |  |
| AT1G69870 | NRT1.7, nitrate transporter 1.7 | HORVU3Hr1G092870 | 49 | -1.52 |  | Low-affinity proton-dependent **nitrate transporter**, Involved in phloem loading and nitrate remobilization from the older leaves to other tissues (Zheng et al., 2016) |
| AT3G45650 | NAXT1, nitrate excretion transporter1 | HORVU7Hr1G025670 | 46 |  | -2.31 | Encodes a **nitrate efflux** transporter NAXT1 (for NITRATE EXCRETION TRANSPORTER1), expressed in the cortex of mature roots (Segonzac et al., 2007). |
| AT5G50200 | NRT3.1, nitrate transmembrane transporters | HORVU5Hr1G115490 | 46 | -2.81 | -3.96 | **Encodes a high-affinity nitrate transporter. Up-regulated by nitrate** (Okamoto et al., 2006; Yong et al., 2010) |
|  |  | HORVU5Hr1G115500 | 46 | -2.53 | -3.82 |  |
|  |  | HORVU6Hr1G053710 | 48 | -1.72 | -1.68 |  |
| AT1G64780 | AMT1;2, ammonium transporter 1;2 | HORVU2Hr1G088380 | 78 | -8.04 | -7.97 | **Ammonium transporter** probably involved in ammonium uptake from the soil (Gazzarrini et al., 1999). |
| AT2G38290  AT2G38290  AT2G38290 | AMT2;1, AMT2, ammonium transporter 2 | HORVU0Hr1G014360 | 69 | -6.23 | -5.74 | **Ammonium transporter 2, Expression in root and shoot is under nitrogen and carbon dioxide regulation** (Neuhäuser et al., 2009)**.** |
|  |  | HORVU1Hr1G070720 | 72 | -3.77 | -5.14 |  |
|  |  | HORVU1Hr1G070730 | 76 | -3.74 | -4.38 |  |
| AT1G20860 | PHT1;8, phosphate transporter 1;8 | HORVU0Hr1G020720 | 55 |  | -1.74 | High-affinity transporter for external **Inorganic phosphate** (Lapis-Gaza et al., 2014; Remy et al., 2012) |
| AT2G38060 | PHT4;2, phosphate transporter 4;2 | HORVU1Hr1G073210 | 66 | -1.65 | -2.16 | **Inorganic phosphate** and probable anion transporter (Irigoyen et al., 2011)**.** |
| AT3G46980 | PHT4;3, phosphate transporter 4;3 | HORVU3Hr1G038250 | 67 | -5.20 | -6.42 | **Inorganic phosphate** and probable anion transporter(Guo et al., 2008) |
| AT2G29650 | ANTR1, phosphate transporter 4;1 | HORVU3Hr1G042770 | 61 | -1.86 | -2.99 | Specific for **Inorganic phosphate** transport across the thylakoid membrane in a sodium dependent manner (Pavón et al., 2008) |
| AT5G44370 | PHT4;6, phosphate transporter 4;6 | HORVU1Hr1G077520 | 67 | -2.98 | -2.85 | **Inorganic phosphate** and probable anion transporter (Hassler et al., 2012) |
|  |  | HORVU3Hr1G103120 | 65 |  | -1.41 |  |
| AT5G43360 | PHT3, PHT1;3, phosphate transporter 1;3 | HORVU4Hr1G002070 | 61 | -4.22 | -1.55 | High-affinity transporter for external **inorganic phosphate** (Hamel et al., 2004; Zhu et al., 2012) |
| AT5G14040 | PHT3;1, phosphate transporter 3;1 | HORVU2Hr1G078800 | 87 | -1.15 | -1.48 | Transport of **phosphate groups** from the cytosol to the mitochondrial matrix. Mediates salt stress tolerance through an ATP-dependent pathway and via modulation of the gibberellin metabolism |
|  |  | HORVU4Hr1G061260 | 75 | -1.23 | -1.28 |  |
| AT5G43350 | PHT1;1, phosphate transporter 1;1 | HORVU5Hr1G110220 | 76 | -6.00 | -9.03 | Phosphate transporter, posttranslational regulation (Bayle et al., 2011; Mudge et al., 2002). |
|  |  | HORVU4Hr1G080350 | 74 | -5.45 | -5.83 |  |
| AT3G48850 | PHT3;2, phosphate transporter 3;2 | HORVU5Hr1G068060 | 66 | -4.46 |  | Transport of **phosphate groups** from the cytosol to the mitochondrial matrix. Mediates salt stress tolerance  (Hamel et al., 2004; Zhu et al., 2012). |
| AT2G17270 | PHT3;3, phosphate transporter 3;3 | HORVU5Hr1G079920 | 66 | -1.76 | -2.97 | **Phosphate transporter**, Response to salt stress (Zhu et al., 2012) |
|  |  | HORVU5Hr1G087910 | 60 | -1.84 | -3.08 |  |
| AT5G20380 | PHT4;5, phosphate transporter 4;5 | HORVU5Hr1G088350 | 69 | -2.51 | -4.04 | **Inorganic phosphate** and probable anion transporter(Guo et al., 2008) |
| AT3G54700 | PHT1;7, phosphate transporter 1;7 | HORVU5Hr1G110180 | 76 | -2.56 | -5.61 | **Phosphate Transporter** (LeBlanc et al., 2013; Mudge et al., 2002) |
| AT3G52190 | PHF1, phosphate transporter traffic facilitator1 | HORVU7Hr1G103510 | 54 | -1.12 | -1.17 | Involved in **phosphate** uptake by facilitating the trafficking of PHT1-1/PHT1;1 from the endoplasmic reticulum to the plasma membrane (González et al., 2005) |
| AT4G22200 | KT2/3, potassium transport 2/3 | HORVU1Hr1G065250 | 66 |  | -3.02 | Highly selective and weak inward-rectifying **potassium channel**. Plays a role in both loading and unloading potassium into/from the phloem sap (Pilot et al., 2003). |
| AT2G40540 | KT2, potassium transporter 2 | HORVU2Hr1G018190 | 73 |  | -1.52 | **Potassium Transporter 2** (Mäser et al., 2001). |
| AT1G60160 | Potassium transporter family protein | HORVU5Hr1G059200 | 67 | -1.91 | -2.35 | **Member of the KT/KUP/HAK family of proton-coupled potassium transporters which have potential effect on cellular expansion** (Ahn et al., 2004) |
| AT5G55630 | KCO1, TPK1, Outward rectifying potassium channel protein | HORVU5Hr1G095540 | 55 | -1.35 | -1.47 | Voltage-independent, large conductance and **potassium-selective** tonoplast ion channel (Gobert et al., 2007). |
|  |  | HORVU5Hr1G095550 | 55 | -5.84 | -6.11 |  |
|  |  | HORVU5Hr1G095590 | 55 | -3.79 | -3.85 |  |
| AT5G14880 | Potassium transporter family protein | HORVU6Hr1G073030 | 68 |  | -1.90 |  |
|  |  | HORVU7Hr1G053250 | 54 | -2.21 | -3.29 |  |
| AT2G30070 | KT1, potassium transporter 1 | HORVU7Hr1G107400 | 53 |  | -6.54 | **Potassium transporter 1**, root growth-sustaining K+ uptake, Osmoticum, and charge carrier (Dennison et al., 2001; Fu & Luan, 1998; Hirsch et al., 1998). |
| AT3G58970 | MGT6, MRS2-4, magnesium transporter 6 | HORVU1Hr1G052760 | 66 | -4.96 | -6.09 | **Transmembrane magnesium transporter. One of nine family members** (Oda et al., 2016) |
| AT1G29820 | Magnesium transporter CorA-like family protein | HORVU3Hr1G060290 | 61 | -1.57 | -2.77 |  |
| AT5G22830 | MGT10, MRS2-11, magnesium (Mg) transporter 10 | HORVU4Hr1G004120 | 79 | -2.25 | -1.81 | High-affinity **magnesium** transporter that mediates the influx of magnesium in chloroplast (Li et al., 2001). |
| AT2G04305 | Magnesium transporter CorA-like family protein | HORVU7Hr1G074050 | 57 | -1.23 |  |  |
| AT1G16010 | MGT2, magnesium transporter 2 | HORVU7Hr1G113400 | 74 | -1.73 | -2.08 | **Magnesium** transporter that may mediate the influx of magnesium (Conn et al., 2011; Lenz et al., 2013). |
| AT4G08620 | SULTR1;1, sulphate transporter 1;1 | HORVU5Hr1G066360 | 71 | -3.06 | -5.33 | High-affinity H^+^/**sulfate** cotransporter that mediates the uptake of the environmental sulfate by plant roots under low-sulfur conditions. Plays a central role in the regulation of sulfate assimilation (Takahashi et al., 2000; Yoshimoto et al., 2002) |
| AT1G30450 | CCC1, ATCCC1, HAP5 \| cation-chloride co-transporter 1 | HORVU5Hr1G090500 | 78 | -2.77 | -3.08 | Cation/chloride cotransporter that mediates potassium-chloride and sodium-**chloride** cotransports. Involved in plant development and Cl^-^ homeostasis. May be involved in long distance Cl^-^ transport (Colmenero-Flores et al., 2007). |
|  |  | HORVU7Hr1G058130 | 82 | -2.13 | -2.40 |  |
|  |  | HORVU7Hr1G058140 | 69 | -8.42 | -8.51 |  |
| AT5G45380 | DUR3, solute:sodium symporters;urea transmembrane transporters | HORVU1Hr1G051450 | 75 | -1.57 | -1.26 | High-affinity urea-proton symporter involved in the active transport of **urea** across the plasma membrane into root cells (Liu et al., 2003) |
| AT3G43660 | Vacuolar iron transporter (VIT) family protein | HORVU2Hr1G101180 | 61 | -3.18 | -2.93 | Probable vacuolar **iron** transporter that may be involved in the regulation of iron distribution throughout the plant |
|  |  | HORVU2Hr1G101310 | 63 | -3.01 | -2.60 |  |
| AT1G76800 | Vacuolar iron transporter (VIT) family protein | HORVU2Hr1G124510 | 69 | -4.86 | -6.32 | Probable vacuolar **iron** transporter involved in the transfer of iron ions from the cytosol to the vacuole for intracellular iron storage (Gollhofer et al., 2014) |
| AT4G19690 | IRT1, iron-regulated transporter 1 | HORVU4Hr1G003050 | 66 | -1.29 |  | **Iron-regulated transporter 1, the gene encodes Fe2+ transporter protein** (Vert et al., 2002) |
| AT4G27870 | Vacuolar iron transporter (VIT) family protein | HORVU7Hr1G003870 | 49 | -2.37 | -2.36 |  |
| AT2G46800 | ZAT, MTP1, ZAT1, zinc transporter | HORVU4Hr1G082000 | 72 | -6.70 | -5.46 | **Zinc transporter**, encodes a member of the cation diffusion facilitator (CDF) and zinc transporter (ZAT) families. It is expressed all across the plant, although it is most noticeable in cells that are proliferating, differentiating, growing and homeostasis (Kawachi et al., 2009). |
|  |  | HORVU1Hr1G015500 | 73 | -1.08 | -1.14 |  |
|  |  | HORVU2Hr1G082080 | 71 | -5.26 | -6.04 |  |
| AT3G12750 | ZIP1, zinc transporter 1 precursor | HORVU4Hr1G073790 | 54 | -3.79 | -5.80 | Mediates **zinc uptake** from the rhizosphere. May also transport copper and cadmium ions (Grotz et al., 1998). |
|  |  | HORVU1Hr1G070450 | 53 |  | -6.59 |  |
|  |  | HORVU2Hr1G025400 | 48 | -3.76 | -4.97 |  |
| AT1G05300 | ZIP5, zinc transporter 5 precursor | HORVU1Hr1G070460 | 51 |  | -3.91 | Probably mediates **zinc uptake** from the rhizosphere. |
|  |  | HORVU4Hr1G073810 | 61 | -4.61 |  |  |
| AT1G31260 | ZIP10, zinc transporter 10 precursor | HORVU4Hr1G003050 | 72 | -1.29 |  | Probably mediates **zinc uptake** from the rhizosphere. |
| AT1G55910 | ZIP11, zinc transporter 11 precursor | HORVU5Hr1G071590 | 55 |  | -1.77 | Probably mediates **zinc uptake** from the rhizosphere. |
| AT1G10970 | ZIP4, zinc transporter 4 precursor | HORVU1Hr1G028920 | 72 | -1.14 | -3.80 | **Zinc transporter 4**, A member of Zrt- and Irt-related protein (ZIP) family. transcript is induced in response to zinc deficiency in the root and shoot (Assunção et al., 2010). |
|  |  | HORVU7Hr1G087490 | 60 | -3.91 | -3.76 |  |
|  |  | HORVU7Hr1G097270 | 59 | -5.23 | -5.32 |  |
| AT5G44790 | RAN1, HMA7, copper-transporting ATPase | HORVU6Hr1G031960 | 75 |  | -1.54 | Involved in **copper import** into the cell. Essential for ethylene signaling, which requires copper. Acts by delivering copper to create functional hormone receptors (Zimmermann et al., 2009) |
|  |  | HORVU7Hr1G108890 | 66 | -1.66 | -2.35 |  |
|  |  | HORVU6Hr1G031970 | 75 |  | -2.11 |  |

**References:**

- Ahn, S. J., Shin, R., & Schachtman, D. P. (2004). Expression of KT/KUP genes in Arabidopsis and the role of root hairs in K+ uptake. *Plant Physiology*, *134*(3), 1135–1145. https://doi.org/10.1104/pp.103.034660
- Assunção, A. G. L., Herrero, E., Lin, Y.-F., Huettel, B., Talukdar, S., Smaczniak, C., Immink, R. G. H., van Eldik, M., Fiers, M., Schat, H., & Aarts, M. G. M. (2010). Arabidopsis thaliana transcription factors bZIP19 and bZIP23 regulate the adaptation to zinc deficiency. *Proceedings of the National Academy of Sciences of the United States of America*, *107*(22), 10296–10301. https://doi.org/10.1073/pnas.1004788107
- Bayle, V., Arrighi, J.-F., Creff, A., Nespoulous, C., Vialaret, J., Rossignol, M., Gonzalez, E., Paz-Ares, J., & Nussaume, L. (2011). Arabidopsis thaliana high-affinity phosphate transporters exhibit multiple levels of posttranslational regulation. *The Plant Cell*, *23*(4), 1523–1535. https://doi.org/10.1105/tpc.110.081067
- Colmenero-Flores, J. M., Martínez, G., Gamba, G., Vázquez, N., Iglesias, D. J., Brumós, J., & Talón, M. (2007). Identification and functional characterization of cation-chloride cotransporters in plants. *The Plant Journal: For Cell and Molecular Biology*, *50*(2), 278–292. https://doi.org/10.1111/j.1365-313X.2007.03048.x
- Conn, S. J., Conn, V., Tyerman, S. D., Kaiser, B. N., Leigh, R. A., & Gilliham, M. (2011). Magnesium transporters, MGT2/MRS2-1 and MGT3/MRS2-5, are important for magnesium partitioning within Arabidopsis thaliana mesophyll vacuoles. *The New Phytologist*, *190*(3), 583–594. https://doi.org/10.1111/j.1469-8137.2010.03619.x
- Deng, W., Luo, K., Li, D., Zheng, X., Wei, X., Smith, W., Thammina, C., Lu, L., Li, Y., & Pei, Y. (2006). Overexpression of an Arabidopsis magnesium transport gene, AtMGT1, in Nicotiana benthamiana confers Al tolerance. *Journal of Experimental Botany*, *57*(15), 4235–4243. https://doi.org/10.1093/jxb/erl201
- Dennison, K. L., Robertson, W. R., Lewis, B. D., Hirsch, R. E., Sussman, M. R., & Spalding, E. P. (2001). Functions of AKT1 and AKT2 potassium channels determined by studies of single and double mutants of Arabidopsis. *Plant Physiology*, *127*(3), 1012–1019.
- Fu, H. H., & Luan, S. (1998). AtKuP1: A dual-affinity K+ transporter from Arabidopsis. *The Plant Cell*, *10*(1), 63–73. https://doi.org/10.1105/tpc.10.1.63
- Gazzarrini, S., Lejay, L., Gojon, A., Ninnemann, O., Frommer, W. B., & von Wirén, N. (1999). Three functional transporters for constitutive, diurnally regulated, and starvation-induced uptake of ammonium into Arabidopsis roots. *The Plant Cell*, *11*(5), 937–948. https://doi.org/10.1105/tpc.11.5.937
- Gobert, A., Isayenkov, S., Voelker, C., Czempinski, K., & Maathuis, F. J. M. (2007). The two-pore channel TPK1 gene encodes the vacuolar K+ conductance and plays a role in K+ homeostasis. *Proceedings of the National Academy of Sciences of the United States of America*, *104*(25), 10726–10731. https://doi.org/10.1073/pnas.0702595104
- Gollhofer, J., Timofeev, R., Lan, P., Schmidt, W., & Buckhout, T. J. (2014). Vacuolar-Iron-Transporter1-Like Proteins Mediate Iron Homeostasis in Arabidopsis. *PLoS ONE*, *9*(10), e110468. https://doi.org/10.1371/journal.pone.0110468
- González, E., Solano, R., Rubio, V., Leyva, A., & Paz-Ares, J. (2005). PHOSPHATE TRANSPORTER TRAFFIC FACILITATOR1 is a plant-specific SEC12-related protein that enables the endoplasmic reticulum exit of a high-affinity phosphate transporter in Arabidopsis. *The Plant Cell*, *17*(12), 3500–3512. https://doi.org/10.1105/tpc.105.036640
- Grotz, N., Fox, T., Connolly, E., Park, W., Guerinot, M. L., & Eide, D. (1998). Identification of a family of zinc transporter genes from Arabidopsis that respond to zinc deficiency. *Proceedings of the National Academy of Sciences of the United States of America*, *95*(12), 7220–7224. https://doi.org/10.1073/pnas.95.12.7220
- Guo, B., Jin, Y., Wussler, C., Blancaflor, E. B., Motes, C. M., & Versaw, W. K. (2008). Functional analysis of the Arabidopsis PHT4 family of intracellular phosphate transporters. *The New Phytologist*, *177*(4), 889–898. https://doi.org/10.1111/j.1469-8137.2007.02331.x
- Hamel, P., Saint-Georges, Y., de Pinto, B., Lachacinski, N., Altamura, N., & Dujardin, G. (2004). Redundancy in the function of mitochondrial phosphate transport in Saccharomyces cerevisiae and Arabidopsis thaliana. *Molecular Microbiology*, *51*(2), 307–317. https://doi.org/10.1046/j.1365-2958.2003.03810.x
- Hassler, S., Lemke, L., Jung, B., Möhlmann, T., Krüger, F., Schumacher, K., Espen, L., Martinoia, E., & Neuhaus, H. E. (2012). Lack of the Golgi phosphate transporter PHT4;6 causes strong developmental defects, constitutively activated disease resistance mechanisms and altered intracellular phosphate compartmentation in Arabidopsis. *The Plant Journal: For Cell and Molecular Biology*, *72*(5), 732–744. https://doi.org/10.1111/j.1365-313X.2012.05106.x
- Hirsch, R. E., Lewis, B. D., Spalding, E. P., & Sussman, M. R. (1998). A role for the AKT1 potassium channel in plant nutrition. *Science (New York, N.Y.)*, *280*(5365), 918–921. https://doi.org/10.1126/science.280.5365.918
- Huang, N. C., Liu, K. H., Lo, H. J., & Tsay, Y. F. (1999). Cloning and functional characterization of an Arabidopsis nitrate transporter gene that encodes a constitutive component of low-affinity uptake. *The Plant Cell*, *11*(8), 1381–1392. https://doi.org/10.1105/tpc.11.8.1381
- Irigoyen, S., Karlsson, P. M., Kuruvilla, J., Spetea, C., & Versaw, W. K. (2011). The sink-specific plastidic phosphate transporter PHT4;2 influences starch accumulation and leaf size in Arabidopsis. *Plant Physiology*, *157*(4), 1765–1777. https://doi.org/10.1104/pp.111.181925
- Kawachi, M., Kobae, Y., Mori, H., Tomioka, R., Lee, Y., & Maeshima, M. (2009). A mutant strain Arabidopsis thaliana that lacks vacuolar membrane zinc transporter MTP1 revealed the latent tolerance to excessive zinc. *Plant & Cell Physiology*, *50*(6), 1156–1170. https://doi.org/10.1093/pcp/pcp067
- Kiba, T., Feria-Bourrellier, A.-B., Lafouge, F., Lezhneva, L., Boutet-Mercey, S., Orsel, M., Bréhaut, V., Miller, A., Daniel-Vedele, F., Sakakibara, H., & Krapp, A. (2012). The Arabidopsis nitrate transporter NRT2.4 plays a double role in roots and shoots of nitrogen-starved plants. *The Plant Cell*, *24*(1), 245–258. https://doi.org/10.1105/tpc.111.092221
- Kim, S. A., Punshon, T., Lanzirotti, A., Li, L., Alonso, J. M., Ecker, J. R., Kaplan, J., & Guerinot, M. L. (2006). Localization of iron in Arabidopsis seed requires the vacuolar membrane transporter VIT1. *Science (New York, N.Y.)*, *314*(5803), 1295–1298. https://doi.org/10.1126/science.1132563
- Kobae, Y., Uemura, T., Sato, M. H., Ohnishi, M., Mimura, T., Nakagawa, T., & Maeshima, M. (2004). Zinc transporter of Arabidopsis thaliana AtMTP1 is localized to vacuolar membranes and implicated in zinc homeostasis. *Plant & Cell Physiology*, *45*(12), 1749–1758. https://doi.org/10.1093/pcp/pci015
- Lapis-Gaza, H. R., Jost, R., & Finnegan, P. M. (2014). Arabidopsis PHOSPHATE TRANSPORTER1 genes PHT1;8 and PHT1;9 are involved in root-to-shoot translocation of orthophosphate. *BMC Plant Biology*, *14*, 334. https://doi.org/10.1186/s12870-014-0334-z
- LeBlanc, M. S., McKinney, E. C., Meagher, R. B., & Smith, A. P. (2013). Hijacking membrane transporters for arsenic phytoextraction. *Journal of Biotechnology*, *163*(1), 1–9. https://doi.org/10.1016/j.jbiotec.2012.10.013
- Lenz, H., Dombinov, V., Dreistein, J., Reinhard, M. R., Gebert, M., & Knoop, V. (2013). Magnesium deficiency phenotypes upon multiple knockout of Arabidopsis thaliana MRS2 clade B genes can be ameliorated by concomitantly reduced calcium supply. *Plant & Cell Physiology*, *54*(7), 1118–1131. https://doi.org/10.1093/pcp/pct062
- Li, L., Tutone, A. F., Drummond, R. S., Gardner, R. C., & Luan, S. (2001). A novel family of magnesium transport genes in Arabidopsis. *The Plant Cell*, *13*(12), 2761–2775. https://doi.org/10.1105/tpc.010352
- Lin, S.-H., Kuo, H.-F., Canivenc, G., Lin, C.-S., Lepetit, M., Hsu, P.-K., Tillard, P., Lin, H.-L., Wang, Y.-Y., Tsai, C.-B., Gojon, A., & Tsay, Y.-F. (2008). Mutation of the Arabidopsis NRT1.5 nitrate transporter causes defective root-to-shoot nitrate transport. *The Plant Cell*, *20*(9), 2514–2528. https://doi.org/10.1105/tpc.108.060244
- Little, D. Y., Rao, H., Oliva, S., Daniel-Vedele, F., Krapp, A., & Malamy, J. E. (2005). The putative high-affinity nitrate transporter NRT2.1 represses lateral root initiation in response to nutritional cues. *Proceedings of the National Academy of Sciences of the United States of America*, *102*(38), 13693–13698. https://doi.org/10.1073/pnas.0504219102
- Liu, L.-H., Ludewig, U., Frommer, W. B., & von Wirén, N. (2003). AtDUR3 encodes a new type of high-affinity urea/H+ symporter in Arabidopsis. *The Plant Cell*, *15*(3), 790–800. https://doi.org/10.1105/tpc.007120
- Maghiaoui, A., Bouguyon, E., Cuesta, C., Perrine-Walker, F., Alcon, C., Krouk, G., Benková, E., Nacry, P., Gojon, A., & Bach, L. (2020). The Arabidopsis NRT1.1 transceptor coordinately controls auxin biosynthesis and transport to regulate root branching in response to nitrate. *Journal of Experimental Botany*, *71*(15), 4480–4494. https://doi.org/10.1093/jxb/eraa242
- Mäser, P., Thomine, S., Schroeder, J. I., Ward, J. M., Hirschi, K., Sze, H., Talke, I. N., Amtmann, A., Maathuis, F. J., Sanders, D., Harper, J. F., Tchieu, J., Gribskov, M., Persans, M. W., Salt, D. E., Kim, S. A., & Guerinot, M. L. (2001). Phylogenetic relationships within cation transporter families of Arabidopsis. *Plant Physiology*, *126*(4), 1646–1667. https://doi.org/10.1104/pp.126.4.1646
- Mayer, M., & Ludewig, U. (2006). Role of AMT1;1 in NH4+ acquisition in Arabidopsis thaliana. *Plant Biology (Stuttgart, Germany)*, *8*(4), 522–528. https://doi.org/10.1055/s-2006-923877
- Mudge, S. R., Rae, A. L., Diatloff, E., & Smith, F. W. (2002). Expression analysis suggests novel roles for members of the Pht1 family of phosphate transporters in Arabidopsis. *Plant Journal*, *31*(3), 341–353. Scopus. https://doi.org/10.1046/j.1365-313X.2002.01356.x
- Muños, S., Cazettes, C., Fizames, C., Gaymard, F., Tillard, P., Lepetit, M., Lejay, L., & Gojon, A. (2004). Transcript profiling in the chl1-5 mutant of Arabidopsis reveals a role of the nitrate transporter NRT1.1 in the regulation of another nitrate transporter, NRT2.1. *The Plant Cell*, *16*(9), 2433–2447. https://doi.org/10.1105/tpc.104.024380
- Narayanan, N., Beyene, G., Chauhan, R. D., Gaitán-Solis, E., Grusak, M. A., Taylor, N., & Anderson, P. (2015). Overexpression of Arabidopsis VIT1 increases accumulation of iron in cassava roots and stems. *Plant Science: An International Journal of Experimental Plant Biology*, *240*, 170–181. https://doi.org/10.1016/j.plantsci.2015.09.007
- Neuhäuser, B., Dynowski, M., & Ludewig, U. (2009). Channel-like NH3 flux by ammonium transporter AtAMT2. *FEBS Letters*, *583*(17), 2833–2838. https://doi.org/10.1016/j.febslet.2009.07.039
- Oda, K., Kamiya, T., Shikanai, Y., Shigenobu, S., Yamaguchi, K., & Fujiwara, T. (2016). The Arabidopsis Mg Transporter, MRS2-4, is Essential for Mg Homeostasis Under Both Low and High Mg Conditions. *Plant & Cell Physiology*, *57*(4), 754–763. https://doi.org/10.1093/pcp/pcv196
- Okamoto, M., Kumar, A., Li, W., Wang, Y., Siddiqi, M. Y., Crawford, N. M., & Glass, A. D. M. (2006). High-affinity nitrate transport in roots of Arabidopsis depends on expression of the NAR2-like gene AtNRT3.1. *Plant Physiology*, *140*(3), 1036–1046. https://doi.org/10.1104/pp.105.074385
- Orsel, M., Chopin, F., Leleu, O., Smith, S. J., Krapp, A., Daniel-Vedele, F., & Miller, A. J. (2007). Nitrate signaling and the two component high affinity uptake system in Arabidopsis. *Plant Signaling & Behavior*, *2*(4), 260–262. https://doi.org/10.4161/psb.2.4.3870
- Pavón, L. R., Lundh, F., Lundin, B., Mishra, A., Persson, B. L., & Spetea, C. (2008). Arabidopsis ANTR1 is a thylakoid Na+-dependent phosphate transporter: Functional characterization in Escherichia coli. *The Journal of Biological Chemistry*, *283*(20), 13520–13527. https://doi.org/10.1074/jbc.M709371200
- Pilot, G., Gaymard, F., Mouline, K., Chérel, I., & Sentenac, H. (2003). Regulated expression of Arabidopsis shaker K+ channel genes involved in K+ uptake and distribution in the plant. *Plant Molecular Biology*, *51*(5), 773–787. https://doi.org/10.1023/a:1022597102282
- Remans, T., Nacry, P., Pervent, M., Filleur, S., Diatloff, E., Mounier, E., Tillard, P., Forde, B. G., & Gojon, A. (2006). The Arabidopsis NRT1.1 transporter participates in the signaling pathway triggering root colonization of nitrate-rich patches. *Proceedings of the National Academy of Sciences of the United States of America*, *103*(50), 19206–19211. https://doi.org/10.1073/pnas.0605275103
- Remy, E., Cabrito, T. R., Batista, R. A., Teixeira, M. C., Sá-Correia, I., & Duque, P. (2012). The Pht1;9 and Pht1;8 transporters mediate inorganic phosphate acquisition by the Arabidopsis thaliana root during phosphorus starvation. *The New Phytologist*, *195*(2), 356–371. https://doi.org/10.1111/j.1469-8137.2012.04167.x
- Segonzac, C., Boyer, J.-C., Ipotesi, E., Szponarski, W., Tillard, P., Touraine, B., Sommerer, N., Rossignol, M., & Gibrat, R. (2007). Nitrate efflux at the root plasma membrane: Identification of an Arabidopsis excretion transporter. *The Plant Cell*, *19*(11), 3760–3777. https://doi.org/10.1105/tpc.106.048173
- Takahashi, H., Watanabe-Takahashi, A., Smith, F. W., Blake-Kalff, M., Hawkesford, M. J., & Saito, K. (2000). The roles of three functional sulphate transporters involved in uptake and translocation of sulphate in Arabidopsis thaliana. *The Plant Journal*, *23*(2), 171–182. https://doi.org/10.1046/j.1365-313x.2000.00768.x
- Tang, H., Vasconcelos, A. C., & Berkowitz, G. A. (1996). Physical association of KAB1 with plant K+ channel alpha subunits. *The Plant Cell*, *8*(9), 1545–1553. https://doi.org/10.1105/tpc.8.9.1545
- Vert, G., Grotz, N., Dédaldéchamp, F., Gaymard, F., Guerinot, M. L., Briat, J.-F., & Curie, C. (2002). IRT1, an Arabidopsis transporter essential for iron uptake from the soil and for plant growth. *The Plant Cell*, *14*(6), 1223–1233. https://doi.org/10.1105/tpc.001388
- Yong, Z., Kotur, Z., & Glass, A. D. M. (2010). Characterization of an intact two-component high-affinity nitrate transporter from Arabidopsis roots. *The Plant Journal: For Cell and Molecular Biology*, *63*(5), 739–748. https://doi.org/10.1111/j.1365-313X.2010.04278.x
- Yoshimoto, N., Takahashi, H., Smith, F. W., Yamaya, T., & Saito, K. (2002). Two distinct high-affinity sulfate transporters with different inducibilities mediate uptake of sulfate in Arabidopsis roots. *The Plant Journal: For Cell and Molecular Biology*, *29*(4), 465–473. https://doi.org/10.1046/j.0960-7412.2001.01231.x
- Zheng, Y., Drechsler, N., Rausch, C., & Kunze, R. (2016). The Arabidopsis nitrate transporter NPF7.3/NRT1.5 is involved in lateral root development under potassium deprivation. *Plant Signaling & Behavior*, *11*(5), e1176819. https://doi.org/10.1080/15592324.2016.1176819
- Zhu, W., Miao, Q., Sun, D., Yang, G., Wu, C., Huang, J., & Zheng, C. (2012). The mitochondrial phosphate transporters modulate plant responses to salt stress via affecting ATP and gibberellin metabolism in Arabidopsis thaliana. *PloS One*, *7*(8), e43530. https://doi.org/10.1371/journal.pone.0043530
- Zimmermann, M., Clarke, O., Gulbis, J. M., Keizer, D. W., Jarvis, R. S., Cobbett, C. S., Hinds, M. G., Xiao, Z., & Wedd, A. G. (2009). Metal binding affinities of Arabidopsis zinc and copper transporters: Selectivities match the relative, but not the absolute, affinities of their amino-terminal domains. *Biochemistry*, *48*(49), 11640–11654. https://doi.org/10.1021/bi901573b
