# Supplementary material for: Comparing apoplastic root barrier formation and morphology in six crop species cultivated in soil vs. hydroponics
Source: Planta. 2025 Nov 1;262(6):141. doi: 10.1007/s00425-025-04862-3 (PMC12579656; doi:10.1007/s00425-025-04862-3)
Supplement: Supplementary file 8 — Supplementary file8 (DOCX 4998 KB) [file 425_2025_4862_MOESM8_ESM.docx]

**Supporting Information**

**Journal Name:** Planta: An International Journal of Plant Biology

**Article title:** Comparing apoplastic root barrier formation and morphology in six crop species cultivated in soil vs. hydroponics

Authors: **Jorge Carvajal^1*#^, Kiran Suresh^1#^, Sabarna Bhattacharyya^2^, Viktoria V. Zeisler-Diehl^1^, Tobias Wojciechowski^3^, Lukas Schreiber^1^**

^1^Department of Ecophysiology, Institute of Cellular and Molecular Botany, University of Bonn, Kirschallee 1, 53115 Bonn, Germany; ^2^Plant Cell Biology, Institute of Cellular and Molecular Botany, University of Bonn, Kirschallee 1, 53115 Bonn, Germany; ^3^Plant Sciences (IBG-2), Forschungszentrum Jülich GmbH, D-52425 Jülich, Germany. ^#^Contributed equally

***Author for correspondence:** Jorge Carvajal

E-Mail: jcar@uni-bonn.de

**The following supporting information is available for this article:**

**Fig. S1:** Phenotypic characterization of hydroponically cultivated and soil-grown plants; **(a)** average root dry weight, **(b)** average shoot dry weight, **(c)** total leaf surface area (cm^2^) **(d)** number of leaves per plant.

**Fig. S2:** Amounts of aliphatic suberin detected among different substance classes in roots grown hydroponically and soil conditions

**Fig. S3:** Amounts of aromatic and total aliphatic suberin detected in roots grown in hydroponics and soil conditions; (a) amount of aromatic suberin, (b) amount of total aliphatic suberin.

**Fig. S4:** Selected DEGs of other genes related to suberin, cutin, cuticular wax and phenylpropanoid biosynthesis commonly upregulated in Zone A roots compared with those in hydroponic zone A and B roots

**Fig. S5:** Selected DEGs of aquaporins commonly up- and downregulated in Zone A of soil-grown roots compared with hydroponic roots in Zones A and B

**Fig. S6:** Gene Ontology (GO) enrichment analysis to identify the top 100 up- and downregulated genes associated with DEGs (FDR < 0.05) of soil-grown ZA versus hydroponically cultivated zones A and B

**Fig. S7:** KEGG pathway analysis of lignin genes via Arabidopsis orthologs (a) Zone A of soil and hydroponics compared with each other, (b) Zone A of soil compared to Zone B of hydroponics compared together.

**Fig. S8:** Selected DEGs of nutrient transporters commonly up- and downregulated in Zone A of soil-grown roots compared with hydroponic roots in Zones A and B.

**Table S1:** List of differentially expressed genes (DEG's) in soil grown root zone A versus hydroponically grown root zone A and root zone B respectively.

**Table S2:** Gene Ontology (GO) enrichment analysis and cross-comparison of differentially expressed genes in barley seminal root soil grown zone A, with hydroponically grown zone A and hydroponically grown zone B root.

**Table S3:** DEGs up- and down-regulated related to suberin, and lignin associated genes with putative barley homologues to their respective Arabidopsis gene ID, its identity percentage, and log2FC.

**Table S4:** DEGs up- and down-regulated related to aquaporins associated genes with putative barley homologues to their respective Arabidopsis gene ID, identity percentage, and log2FC.

**Table S5:** Chemical composition of the cultivation conditions.

**Table S6:** Top 100 genes up/down-regulated list with Log2FC and FDR, and Gene Ontology (GO) enrichment analysis.

**Table S7:** DEGs up- and down-regulated related to nutrient transporters associated genes with putative barley homologues to their respective Arabidopsis gene ID, its identity percentage, and log2FC.

**Fig. S1** Phenotypic characterization of hydroponically cultivated and soil-grown plants. **a** Average root dry weight. **b** Average shoot dry weight. **c** Total leaf surface area. **d** Number of leaves per plant. **a**, **b** and **c** The box ranges from the 25th to 75th percentiles, and the square inside the box represents the mean value. The whiskers range to outliers, and each box represents >6 individual plant measurements. Each crop species was separately analyzed via one-way ANOVA (Fischer’s least significant difference, LSD), with the medium of growth as a factor. Significance: *P* < 0.001**; *P* < 0.01**; *P* < 0.05*; n.s. = not significant. **d** The bar represents the mean number of leaves


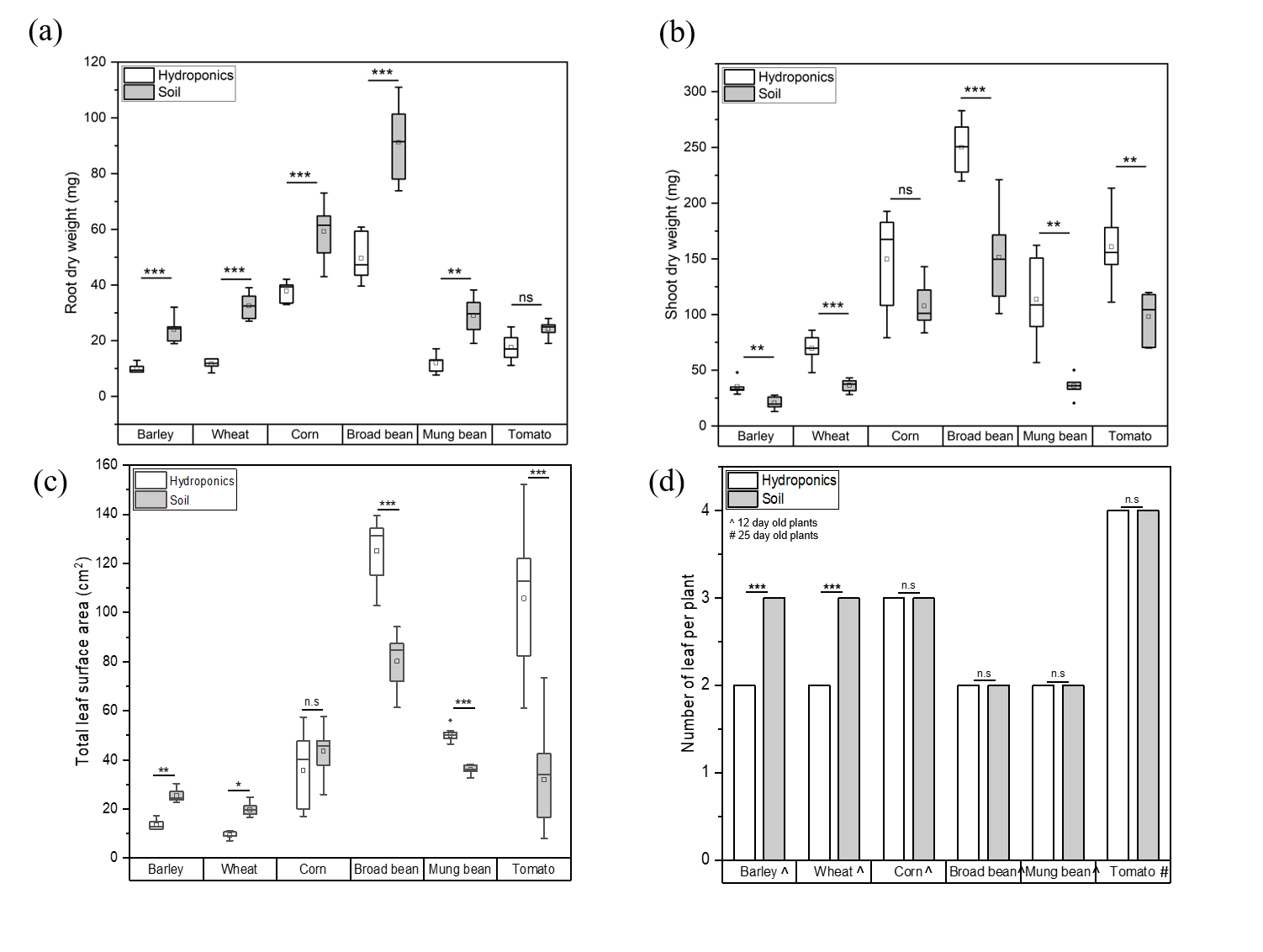


**Fig. S2** Amounts of aliphatic suberin detected among different substance classes in roots grown hydroponically and soil conditions. The roots were divided into three root zones, from root tip Zone A, Zone B, and Zone C, toward the basal part. The bars represent the mean values with a standard deviation of at least three biological replicates (*n* = 3). Different letters indicate significant differences between the means at a significance level of 0.05 according to one-way ANOVA (Fischer’s least significant difference, LSD). The significance level was tested for different root zones within the same species. The predominant substance classes in monocots are ω-OH and diacids. In dicots, more fatty acids can be detected in broad bean and mung bean, along with ω-OH and diacids. FA = fatty acids; Alc = alcohols; ω-OH = hydroxyacids


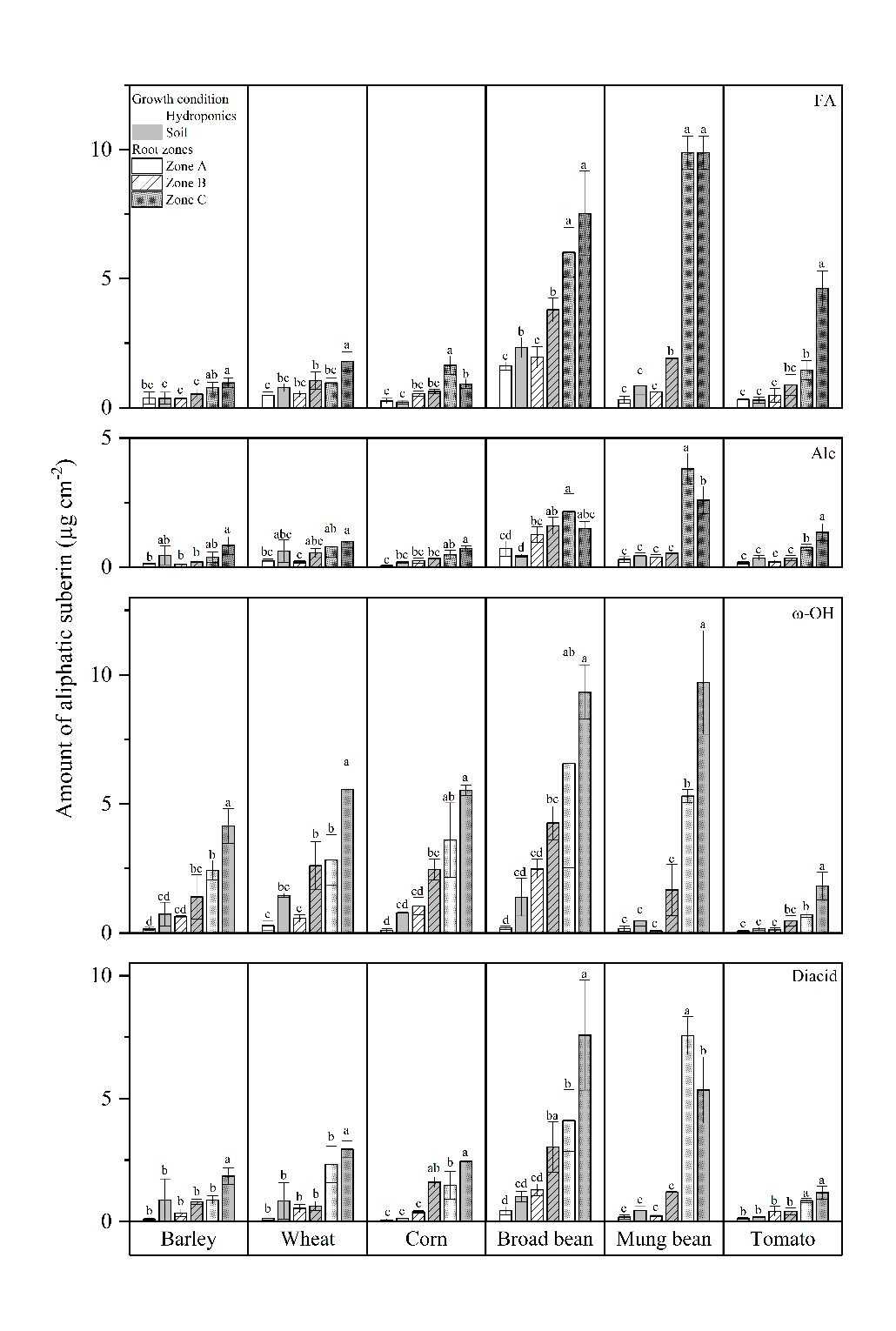


**Fig. S3** **a** Amounts of aromatic suberin detected in roots grown hydroponically and soil conditions. **b** Amounts of total suberin (sum of aromatic and aliphatic amounts) detected in roots grown hydroponically and soil conditions. The roots were divided into three root zones from root tip Zones A, B, and C toward the basal part. The bars represent the mean values with a standard deviation of at least three biological replicates (*n* = 3). Different letters indicate significant differences between the means at a significance level of 0.05 according to one-way ANOVA (Fischer’s least significant difference, LSD). The significance level was tested for different root zones within the same species. The amount of total and aromatic suberin tended to increase across the root zones from A to C under both hydroponic and soil conditions. Most of the soil-grown roots contained more suberin than did the roots of hydroponically cultivated plants


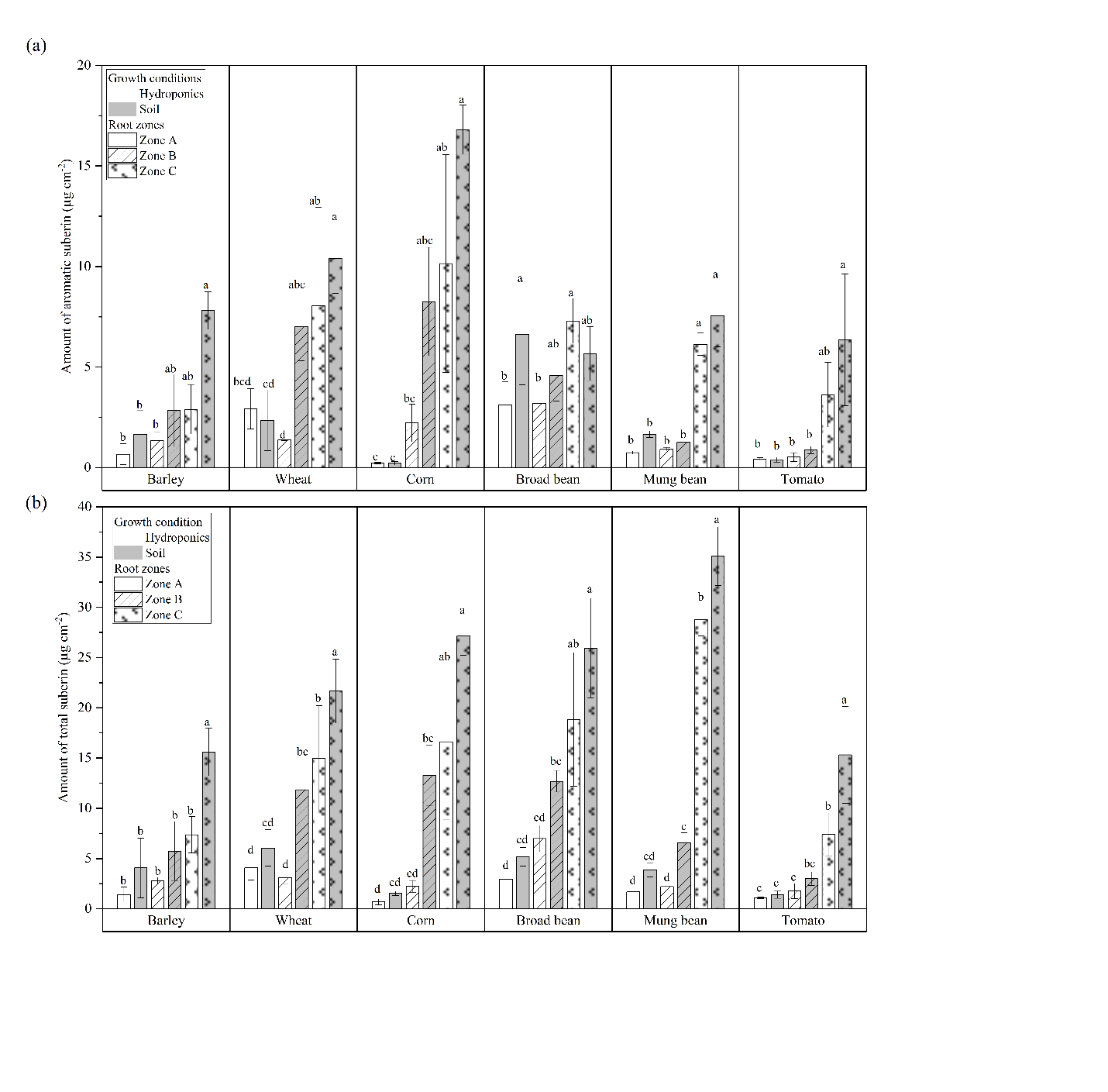


**Fig. S4** Selected DEGs of other genes related to suberin, cutin, cuticular wax and phenylpropanoid biosynthesis commonly upregulated in Zone A of soil-grown roots compared with those of hydroponically grown Zone A and B roots. Note: Empty white cells are not significant (n.s.)


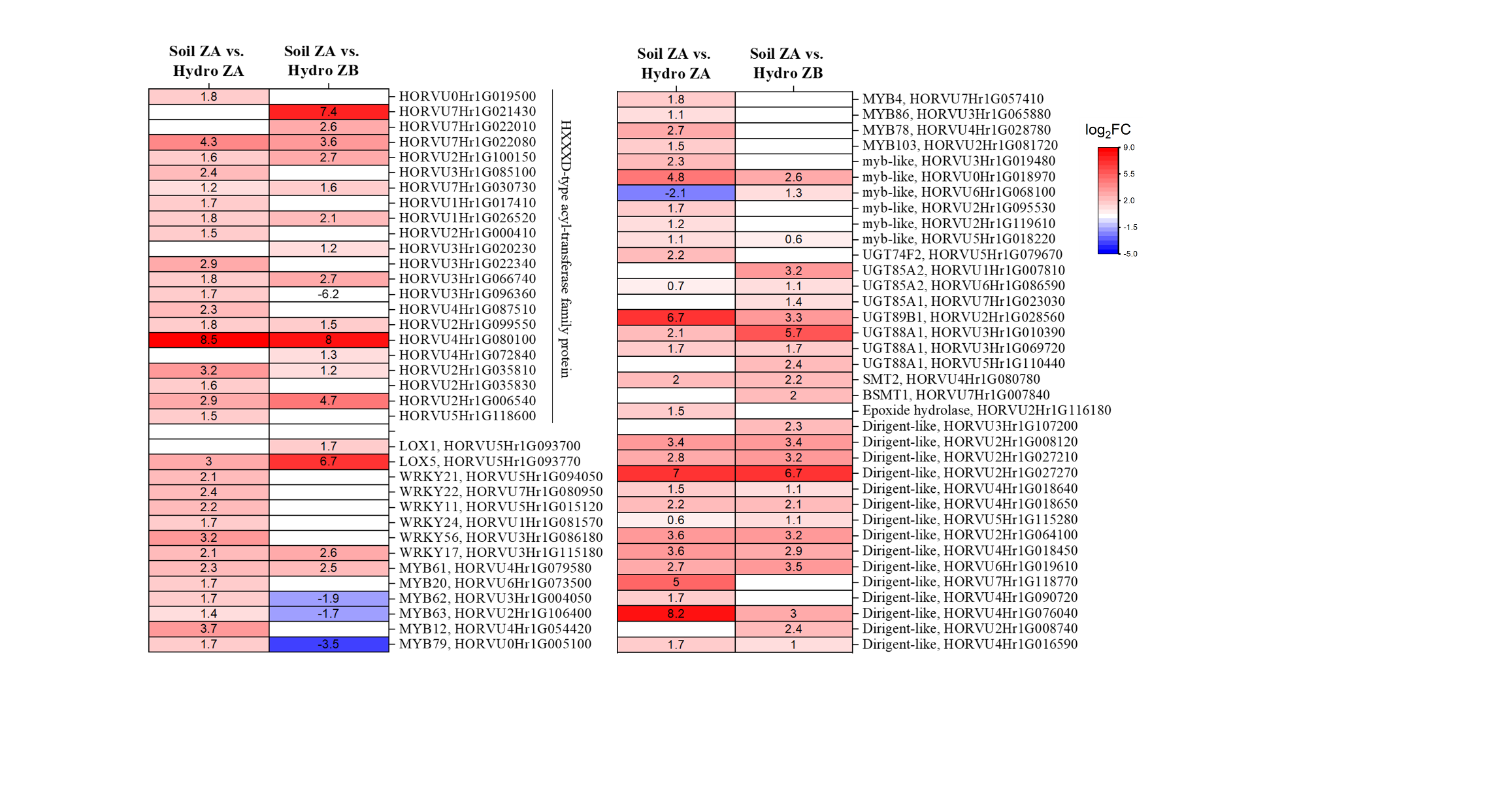


**Fig. S5** Selected DEGs of aquaporins commonly up- and downregulated in Zone A of soil-grown roots compared with hydroponically grown roots in Zones A and B. Note: Empty white cells are not significant (n.s.). Genes with putative barley homologs to their respective Arabidopsis gene ID, identity percentage, log2FC, description and references are given in Table S4

roo
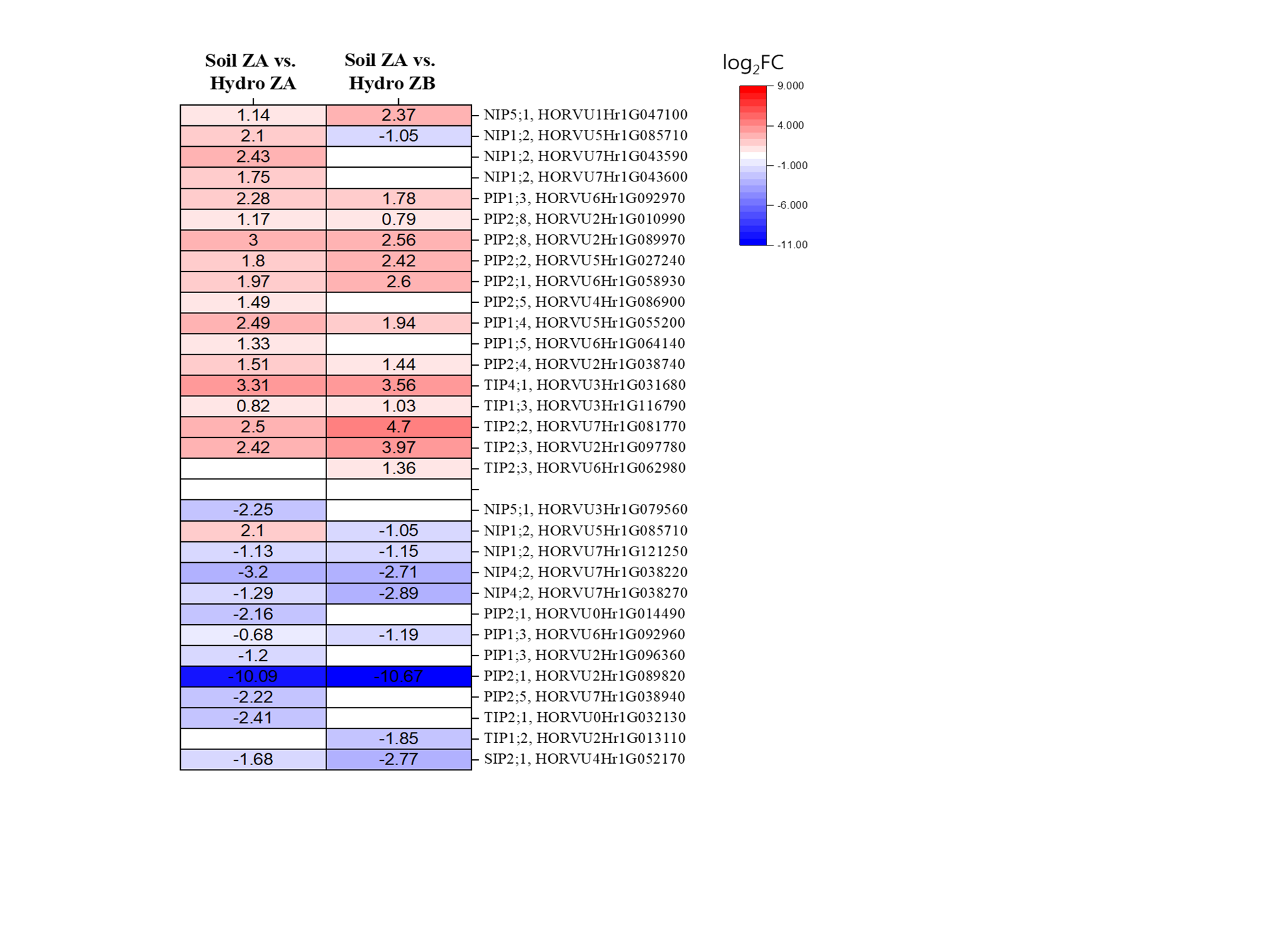


**Fig. S6** Gene Ontology (GO) enrichment analysis to identify the top 100 up- and downregulated genes associated with DEGs (FDR < 0.05) of soil-grown roots of Zone A (ZA) versus hydroponically cultivated roots of Zones A and B (ZB). These genes were selected by FDR and sorted by fold enrichment. Upregulated (**a, b**) and downregulated (**c, d**) genes. **a** Top 100 upregulated genes in soil Zone A compared with those in hydroponics Zone A; **b** top 100 upregulated genes in soil Zone A compared with those in hydroponics Zone B; **c** top 100 downregulated genes in soil Zone A compared with those in hydroponics Zone A; **d** top 100 downregulated genes in soil Zone A compared with those in hydroponics Zone B. Table S3 lists the genes, the fold change values, and other GO term pathways in detail


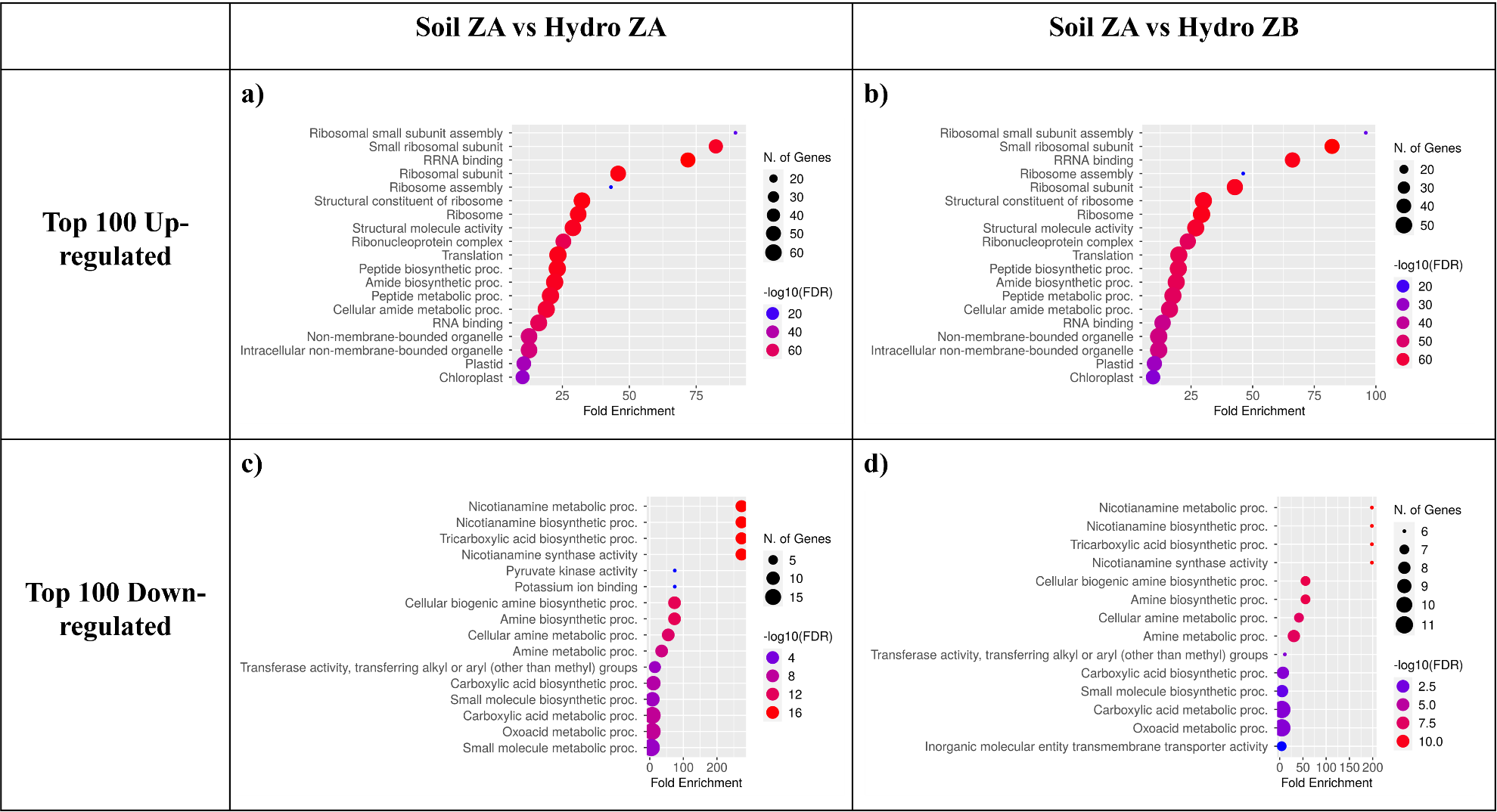


**Fig. S7** KEGG pathway analysis of lignin genes via Arabidopsis orthologs. The genes PAL2, HCT, FAH1, CYP84A1, OMT1, PRX17, PRX25, PRX52, PRX72, and PER39 are presented as red boxes. These lignin genes are upregulated in soil-grown roots compared with those in hydroponically grown roots. **a** Zone A of the roots cultivated in soil, and Zone A of the roots in hydroponic solution are compared. **b** Zone A of the soil and Zone B of the hydroponic solution are compared.


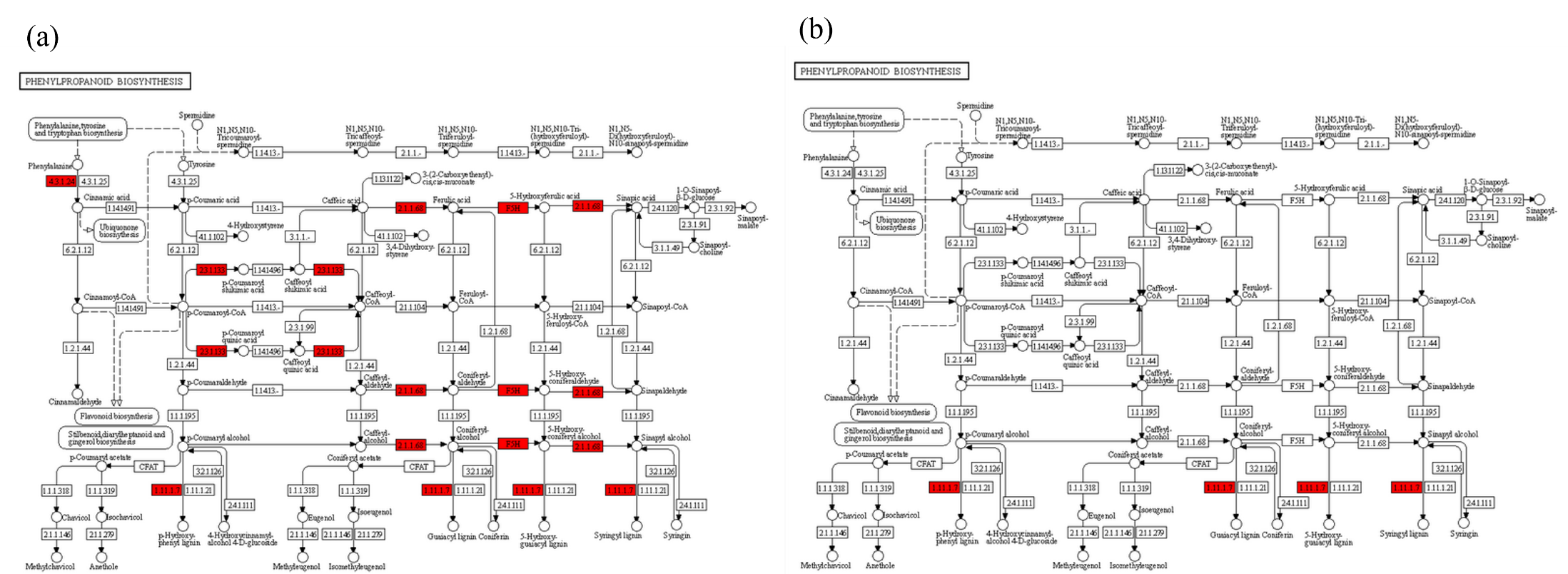
**Fig. S8** Selected DEGs of nutrient transporters commonly up- and downregulated in Zone A of soil-grown roots compared with hydroponically grown roots in Zones A and B. Note: Empty white cells are not significant (n.s.). Genes with putative barley homologs to their respective Arabidopsis gene ID, identity percentage, log2FC, description and references are given in Table S7.


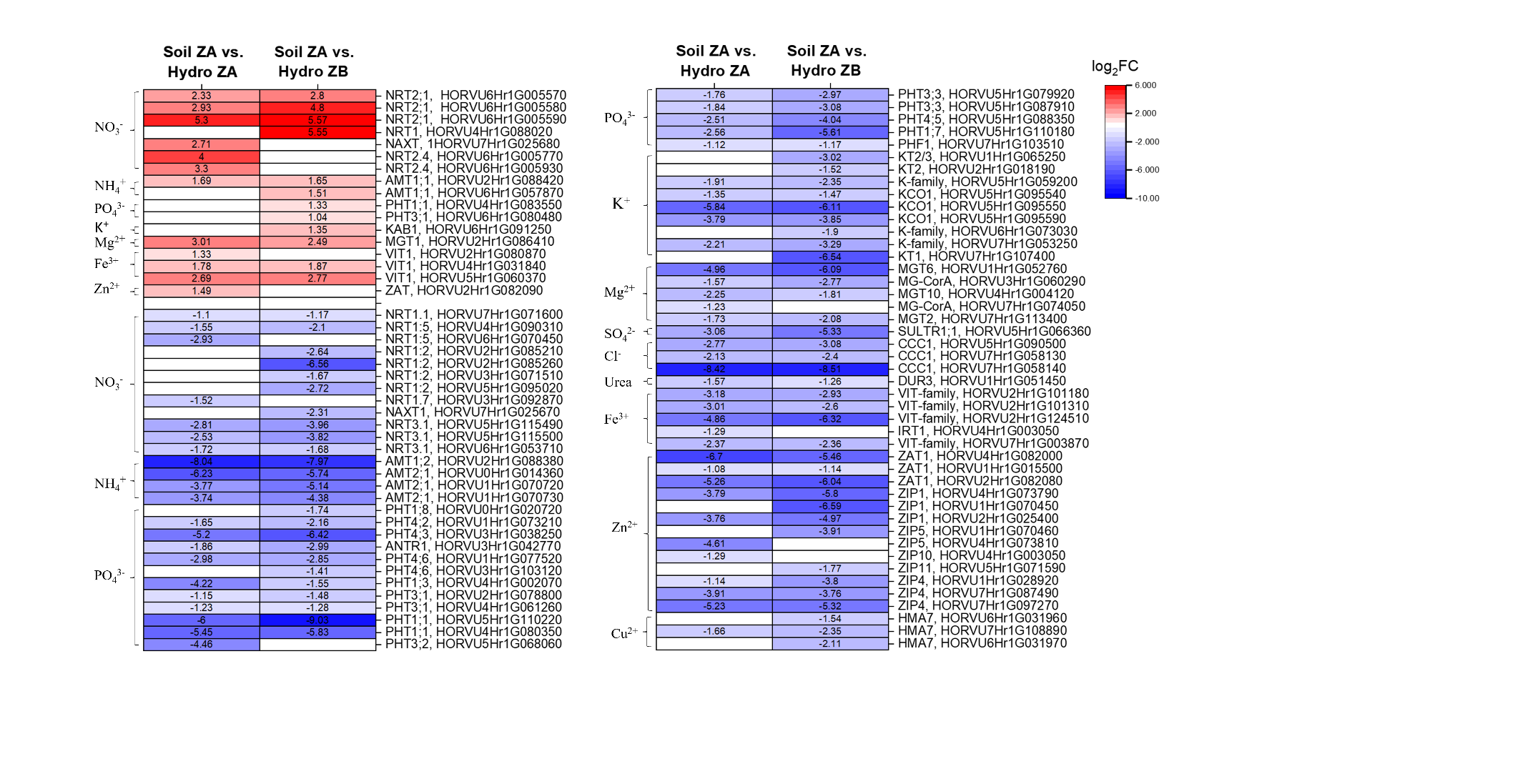


**Note:** Each Excel file has an info sheet at the beginning with all the details.

**Table S1** Complete list of differentially expressed genes (DEG’s) in soil grown root zone A versus hydroponically grown root zone A and root zone B respectively.

Sheets: GeneID, log2FC, Average and standard deviation of CPM list are also given here.

**Table S2** Gene Ontology (GO) enrichment analysis and cross-comparison of differentially expressed genes in the barley seminal root soil grown zone A with hydroponically grown zone A and hydroponically grown zone B root. The GO terms are obtained in shinyGO after adding the list of genes obtained from the venn diagram (Figure 6c)

Sheets: Commonly UP-Regulated in both the regions: (Soil Zone A vs Hydroponics Zone A) & (Soil Zone A vs Hydroponics Zone B)

Sheets: Only UP-Regulated in ZA: (Soil Zone A vs Hydroponics Zone A)

Sheets: Only UP-Regulated in ZB: (Soil Zone A vs Hydroponics Zone B)

Sheets: Commonly DOWN-Regulated in both the regions: (Soil Zone A vs Hydroponics Zone A) & (Soil Zone A vs Hydroponics Zone B)

Sheets: Only Down-Regulated in ZA: (Soil Zone A vs Hydroponics Zone A)

Sheets: Only Down-Regulated in ZB: (Soil Zone A vs Hydroponics Zone B)

**Table S3** DEGs up- and down-regulated related to suberin, and lignin associated genes with putative barley homologues to their respective Arabidopsis gene ID, its identity percentage, **and log2FC. The list is given for both Soil zone A vs. Hydroponics zone A and Soil zone A vs. Hydroponics zone B, and references for the description of each gene are also given.**

**Table S4** DEGs up- and down-regulated related to aquaporins associated genes with putative barley homologues to their respective Arabidopsis gene ID, its identity percentage, **and log2FC. The list is given for both Soil zone A vs. Hydroponics zone A and Soil zone A vs. Hydroponics zone B, and references for the description of each gene are also given.**

**Table S5** Chemical composition of the two cultivation conditions.

**Table S6** Top 100 genes up/down-regulated list with log2FC and FDR are given along with Gene ontology (GO) enrichment analysis for these 100 genes.

Sheets: Top 100 up-regulated in Soil grown zone A vs Hydroponically grown zone A

Sheets: Top 100 up-regulated in Soil grown zone A vs Hydroponically grown zone B

Sheets: Top 100 down-regulated in Soil grown zone A vs Hydroponically grown zone A

Sheets: Top 100 down-regulated in Soil grown zone A vs Hydroponically grown zone B

**Table S7** DEGs up- and down-regulated related to nutrient transporters associated genes with putative barley homologues to their respective Arabidopsis gene ID, its identity percentage, **and log2FC. The list is given for both Soil zone A vs. Hydroponics zone A and Soil zone A vs. Hydroponics zone B, and references for the description of each gene are also given.**
